# Supplementary material for: Effects of transcranial direct current stimulation (tDCS) and concurrent cognitive training on episodic memory in patients with traumatic brain injury: a double-blind, randomised, placebo-controlled study
Source: BMJ Open. 2021 Aug 26;11(8):e045285. doi: 10.1136/bmjopen-2020-045285 (PMC8395342; doi:10.1136/bmjopen-2020-045285)
Supplement: Author's manuscript [file bmjopen-2020-045285.draft_revisions.pdf]

BMJ Open is committed to open peer review. As part of this commitment we make the peer review history of every article we publish publicly available.

When an article is published we post the peer reviewers' comments and the authors' responses online. We also post the versions of the paper that were used during peer review. These are the versions that the peer review comments apply to.

The versions of the paper that follow are the versions that were submitted during the peer review process. They are not the versions of record or the final published versions. They should not be cited or distributed as the published version of this manuscript.

BMJ Open is an open access journal and the full, final, typeset and author-corrected version of record of the manuscript is available on our site with no access controls, subscription charges or pay-per-view fees (http://bmjopen.bmj.com).

If you have any questions on BMJ Open's open peer review process please email info.bmjopen@bmj.com

BMJ Open

# The effects of transcranial direct current stimulation (tDCS) and cognitive training on episodic memory in patients with traumatic brain injury: a double-blind, randomized, placebo-controlled study

| | |
|---|---|
| Journal: | *BMJ Open* |
| Manuscript ID | bmjopen-2020-045285 |
| Article Type: | Protocol |
| Date Submitted by the Author: | 26-Sep-2020 |
| Complete List of Authors: | DE FREITAS, DAGLIE; HCFMUSP, Neurology/Neurosurgery<br>Zaninotto, Ana Luiza; MGH Institute of Health Professions, Speech and Feeding Disorders Lab<br>Brunoni, Andre; University of São Paulo, Division of Psychiatry and Psycology<br>Paiva, Wellingson; University of São Paulo, Division of Neurosurgery<br>De Carvalho, Daniel; HCFMUSP, Neurology/Neurosurgery<br>Paglioni, Vanessa Maria; HCFMUSP, Neurology/Neurosurgery<br>Souza, Maria Sigride; University of Sao Paulo, Psychiatry Department<br>Valiengo, Leandro; University of Sao Paulo, Interdisciplinary Center for Applied Neuromodulation and Service of Interdisciplinary Neuromodulation<br>Guirado, Vinícius; HCFMUSP, Division of Neurosurgery |
| Keywords: | Neurological injury < NEUROLOGY, Rehabilitation medicine < INTERNAL MEDICINE, Clinical trials < THERAPEUTICS |
| | |

SCHOLARONE™
Manuscripts

*I, the Submitting Author has the right to grant and does grant on behalf of all authors of the Work (as defined in the below author licence), an exclusive licence and/or a non-exclusive licence for contributions from authors who are: i) UK Crown employees; ii) where BMJ has agreed a CC-BY licence shall apply, and/or iii) in accordance with the terms applicable for US Federal Government officers or employees acting as part of their official duties; on a worldwide, perpetual, irrevocable, royalty-free basis to BMJ Publishing Group Ltd ("BMJ") its licensees and where the relevant Journal is co-owned by BMJ to the co-owners of the Journal, to publish the Work in this journal and any other BMJ products and to exploit all rights, as set out in our licence.*

*The Submitting Author accepts and understands that any supply made under these terms is made by BMJ to the Submitting Author unless you are acting as an employee on behalf of your employer or a postgraduate student of an affiliated institution which is paying any applicable article publishing charge ("APC") for Open Access articles. Where the Submitting Author wishes to make the Work available on an Open Access basis (and intends to pay the relevant APC), the terms of reuse of such Open Access shall be governed by a Creative Commons licence – details of these licences and which Creative Commons licence will apply to this Work are set out in our licence referred to above.*

*Other than as permitted in any relevant BMJ Author's Self Archiving Policies, I confirm this Work has not been accepted for publication elsewhere, is not being considered for publication elsewhere and does not duplicate material already published. I confirm all authors consent to publication of this Work and authorise the granting of this licence.*

For peer review only

**The effects of transcranial direct current stimulation (tDCS) and cognitive training on episodic memory in patients with traumatic brain injury: a double-blind, randomized, placebo-controlled study**

Dagliê Jorge de Freitas [1], Daniel de Carvalho[1], Vanessa M. Paglioni[1], Andre R. Brunoni[2,3], Leandro Valiengo[2,3], Sigride Thome-Souza[4], Vinicius Monteiro de Paula Guirado[1], Ana Luiza Zaninotto[1,5]*, Wellingson S. Paiva[1]*

*Sharing the last authorship.

1. Department of Neurology/ Neurosurgery, Clinics Hospital, Medical School of University of Sao Paulo (HC-FMUSP), Brazil.
2. Interdisciplinary Center for Applied Neuromodulation and Service of Interdisciplinary Neuromodulation, LIM-27, University of São Paulo, Brazil
3. Departments of Internal Medicine and Psychiatry, Faculdade de Medicina da Universidade de São Paulo, Brazil
4. Service of video-electroencephalography, Psychiatry Department, School of Medicine, University of Sao Paulo, Sao Paulo, Brazil.
5. Speech and Feeding Disorders Lab, MGH Institute of Health Professions (MGHIHP), Boston, USA.

Word count: 2918

Corresponding author:
Ana Luiza Zaninotto
79/96 13th street, 2nd floor.
Boston, MA, United States
02129
Correspondence: acostazaninotto@mghihp.edu

For peer review only

## ABSTRACT

**Introduction:** Deficits in episodic memory following traumatic brain injury (TBI) are common and affect independence in activities of daily living. Concomitant transcranial direct current stimulation (tDCS) and cognitive outcomes may contribute to improve episodic memory in patients with TBI. Although previous studies have shown the potential benefits of tDCS to improve cognition, the benefits of the tDCS applied concomitantly with cognitive training are still inconsistent. This study aims to (1) investigate whether active tDCS combined with computer-assisted cognitive training enhances episodic memory compared to sham tDCS; (2) to compare the differences between active tDCS applied over the left dorsolateral prefrontal cortex (lDLPFC) and bilateral temporal cortex (BTC) on episodic memory; and (3) investigate inter and intra-group changes on cortical activity measured by quantitative electroencephalogram (qEEG).

**Methods and analysis:** A randomized, parallel-group, double-blind placebo-controlled study is conducted. Thirty-six participants with chronic, moderate, and severe closed TBI are being recruited. Participants are randomized into three parallel groups (1:1:1) based on the placement of tDCS sponges and activation (active or sham). TDCS is applied for 10 consecutive days for 20 minutes, concomitant to a computer-based memory and attention training. Cognitive scores and qEEG are collected at baseline, on the last day of the stimulation session, and 3 months after the last tDCS session. Based on previous studies we hypothesize that (1) memory scores in the active tDCS group will improve compared to the sham group; (2) memory scores will be higher after the BTC active tDCS compared to the lDLPFC; (3) there will be significant delta reduction and an increase in alpha waves close to the location of the active sponge placement compared to the sham group.

**Ethics and dissemination:** This study was approved by Hospital das Clínicas, University of São Paulo Ethical Institutional Review Border (number CAAE: 87954518.0.0000.0068)

**Trial registration number:** ClinicalTrials.gov(NCT04540783).


**Keywords:** traumatic brain injury, episodic memory, transcranial direct current stimulation, cognition, rehabilitation.

For peer review only

## ARTICLE SUMMARY

### Strengths and limitations of this study

- This protocol is the first randomized controlled trial investigating the effects of the concomitant transcranial direct current stimulation (tDCS) and computer-assisted cognitive training on episodic memory in individuals with TBI.
- This study may contribute to the development of evidence-based low-risk and low-cost rehabilitation treatments for TBI survivors with memory impairments.
- The location of the brain injury may bias the results, however, since all the participants also have diffuse axonal injury, we expect that the tDCS will act in a similar manner across the participants.

For peer review only

## INTRODUCTION

Traumatic Brain Injury (TBI) is an alteration in brain function caused by an external force and a major cause of death and disability throughout the world. (1,2) The hippocampus and the prefrontal cortex are among the brain structures more susceptible to lesions after a brain insult and, as a consequence, head injury survivors may experience difficulties in recalling specific events from the personal past and imagining novel scenarios. (3–5) Those regions are known to play important roles in episodic memory, which is a declarative memory containing information about place and time of acquisition as opposed to semantic memory, which refers to memory not tied to the context of encoding. (6) The hippocampus specifically organizes experienced and biographic memories that are defining features of episodic memory, and the pre-frontal cortex suppresses context-inappropriate memories thus allowing the retrieval of context-appropriate memories. (7) After brain trauma, cognitive impairment might be persistent (8) and no treatments available have been shown to be effective to improve those sequels.

Non-invasive brain stimulation (NIBS) techniques, including transcranial direct current stimulation (tDCS), are neuromodulatory interventions that have been shown to improve neuroplasticity and cognitive outcomes in neurological conditions, including TBI. (9,10) tDCS can transiently alter neuronal activity facilitating or inhibiting neuronal circuitries depending on the polarity of the stimulation. (11) tDCS induces neuroplasticity by applying a low-intensity electrical current (0.5 - 2 mA) through electrodes placed on the scalp. The electrodes have two polarities (anode and cathode) and change the resting state of the membrane cells of the surrounding region. (12,13) Previous studies have shown that repetitive tDCS sessions improved disorder of consciousness (11,14,15) and cognition in patients with TBI, (8,16) despite some studies have shown inconsistent results. (17–19)

For peer review only

Since tDCS works on the membrane level, changing the resting state but not evoking action potential, the use of cognitive training concomitant to the tDCS seems to be a good option to potentiate the stimulation and modulate the brain networks accordingly to the target training. (20–23) Two prior studies investigated the effects of the concomitant use of tDCS and offline cognitive training on memory and attention performance in TBI patients, but only one found a significant improvement in the cognitive outcome measures. (20,22)

Biomarkers that evaluate brain changes after the tDCS intervention are still scarce, however the electroencephalogram (EEG) has been suggested to be a useful tool for this purpose. (24–29) The EEG measures the rhythm of electrical activity in the brain according to its frequency: Delta (1 - 4Hz), Theta (4 -8Hz), Alpha (8 - 12Hz) and Beta (12 - 30Hz) and Gamma (30 - 40Hz) (27,30,31) and is widely used as a safety outcome in patients who undergo tDCS sessions. (32–35) Some studies associate EEG measures (amplitude, power, phase and coherence) to the functionality of patients, (36) including the diagnosis and prognosis of patients with TBI. (37,38) A study using EEG power spectrum (39) suggests that, after 10 tDCS sessions, changes in the rhythm of brain activity occur, with reduction of delta and increase of alpha near the active electrodes in patients with chronic TBI. This study also found a significant correlation between decreases in delta and improved performance on neuropsychological tests for the active tDCS group to far greater extent than for the sham group. (24) Other studies have measured cortical activity after a single session of tDCS and have shown inconsistent results. (24–29) Thus, cortical changes after consecutive sessions of tDCS with concomitant cognitive training in people with TBI are still inconclusive.

Due to the lack of consensus and scarcity of evidence about the effects of cognitive training in addition to tDCS sessions in patients with TBI, the goals of this study are (a)

For peer review only

to investigate the effect of 10 sessions of concomitant tDCS and online cognitive training in patients with TBI compared to sham tDCS; (b) to analyze differences on episodic memory scores between active tDCS over lDLPF and BTC; (c) to analyze changes on cortical activities (measured by the EEG) between the groups. We hypothesize that (a) participants that received active stimulation will have greater scores on episodic memory test compared to the sham group; (b) active tDCS over the BTC will demonstrate higher episodic memory scores compared to the lDPFC; (c) delta reduction and an increase in alpha waves close to the of sponge placement in the active group compared to the sham group.

## METHODS AND ANALYSIS

### Design

This study is a randomized, parallel-group, placebo-controlled and double-blind study that is being conducted at Hospital das Clínicas, University of São Paulo, (HC-FMUSP), São Paulo, Brazil. Participants who meet eligibility criteria are randomly allocated to (1) Group 1 - bilateral temporal cortex (BTC); (2) Group 2- left dorsolateral prefrontal cortex (lDLPFC); (3) Group 3 – sham (BTC/lDLPFC).

Twenty-minute-tDCS for 10 days (2 weeks, except for Saturdays and Sundays) is delivered concomitant to the cognitive training. Patients will be assessed at baseline (T0), at the end of the last stimulation session (T1) and three months after the last tDCS session (T2). (Figure 1).

### Ethics committee and regulatory approval

The trial is conducted in accordance with the ethical principles outlined in the Declaration of Helsinki, 1996. This research was approved by the Hospital das Clínicas, University of São Paulo Ethical Institutional Review Border number CAAE:

For peer review only

87954518.0.0000.0068. Clinical Trial number NCT04540783. Any severe side effect during the trial will be reported to the safety monitoring board IRB for appropriate management.

**Randomization and blinding**

The investigator ALZ was responsible for the computer-generated random assignment list, arranging patients in blocks of 3 or 6. The proportion of the randomization for each group is 1:1:1. This randomized list ensures double blinding so that both research assistants and patients are blind to the type of stimulation. Before each stimulation session, the researcher responsible for the stimulation receives a code that allows the tDCS device to deliver 20 minutes of active or sham stimulation. This blinding and methodological procedure is similar to the rational of previous studies. (20,22,24)

The randomization list and the Neuroconn (tDCS device) code is kept inside a locked drawer with restricted access at the research coordination office at Hospital das Clínicas (HC-FMUSP).

**Recruitment and study population**

Thirty-six patients with TBI are being recruited from hospitals in São Paulo. All participants provide written informed consent and receive an exclusive identification number during the screening period, to ensure blinding. Study recruitment started in June 2019 and the estimated completion date for the primary outcome is June 2022. We expect that 85% of the patients will be inpatients from HC-FMUSP referred by neurologists and 15% from extra-mural recruitment (from social media and folders). This trial follows the CONSORT (Consolidated Standards of Reporting Trials) guidelines.

For peer review only

**Inclusion criteria**

• Outpatients with radiological diagnosis of TBI at least 6 months prior to enrollment in the study.

• Glasgow Coma Scale (GCS) score ≤ 12 at admission in the emergency room.

• Memory complaints, self-reported or reported by the family/ caregiver-.

• Age between 18 and 60 years.

• Able to follow directions.

**Exclusion criteria**

• History of epilepsy post-TBI.

• Clinical EEG abnormalities (epileptiform activity, disorganized background , in other words, a general change in the way a normal brain wave looks – frequency, height and shape).

• Uncorrected visual impairment.

• Contraindications to tDCS, such as medical devices implanted in the brain or metallic foreign body in the head.

• Current severe/major depression (score over 36 points on the Beck Depression Inventory – 2nd edition).

• Current severe anxiety (score over 26 points on the Back Anxiety Inventory).

• Limiting motor deficit.

• Estimated IQ under 70.

**Patient and public involvement**

Patients are not involved in the design, or conduct, or reporting, or dissemination plans of our research.

For peer review only

**Instruments**

Patients are expected to come to the research hospital for 11 visits as described in Table 1.

Table 1: Detail of the study visits.

| | Tasks | Visit 1 Baseline | Visit 2-9 | Visit 10 | Visit 11 3 months follow-up |
|---|---|---|---|---|---|
| Consent Form | X | | | | |
| Screening | | | | | |
| Medical history | X | | | | |
| qEEG | X | | | X | X |
| BDI-II | X | | | X | X |
| BAI | X | | | X | X |
| Estimated IQ | X | | | | |
| Episodic Memory | | | | | |
| RAVLT | | X | | X | X |
| Intervention | | | | | |
| tDCS | | X | X | X | |
| Cognitive Training | | X | X | X | |
| AEQ | | X | X | X | |

Legend: AEQ – Adverse Events Questionnaire; BAI - Beck Anxiety Inventory; BDI-II - Beck Depression Inventory; qEEG – Quantitative Electroencephalogram; IQ – Intelligence quotient, tDCS – Transcranial Direct Current Stimulation, RAVLT - Rey Auditory Verbal Learning Test.

**Screening assessment:**

Depressive symptoms - BDI-II - Beck Depression Inventory (40)

Anxiety symptoms - BAI - Beck Anxiety Inventory (41)

Estimated IQ - WAIS - Wechsler Adult Intelligence Scale (Matrix Reasoning and Vocabulary) (42,43).

**Primary outcome (Episodic Memory)**

Rey Auditory Verbal Learning Test (RAVLT) - A list of 15 words is presented, and subjects are asked to say all words they can remember. The process is repeated 5 times. Twenty minutes after the 5th trial, an interference list of 15 words (List B) is presented, followed by a free-recall test of that list. Immediately after this, delayed recall of the first list is tested (Trial 6) without further presentation of those words. After a 20-

For peer review only

min delay period, subjects are again required to recall words from List A (Trial 7). Finally, recognition can be tested with the use of a matrix array, in which the individual must identify List A words from a list of 50 words containing all items from Lists A and B and 20 words that are phonemically or semantically similar to those in Lists A and B. (44)

**Secondary outcome**

EEG assessment. The exam is performed on the Nihon Kohden® EEG 1200 version 01.71 digital equipment, with simultaneous video recording with a Sony® Ipela camera. For qualitative EEG data collection, we use the international 10-20 electrode placement system, 19 channels (being one to electrocardiogram), with sampling rate of 200Hz, a time of 0.3, high filter from 35 to 70 Hz and sensitivity of 7 µV. For the quantitative analysis, the data are converted using Neuromap from the Neuroworkbench software. The exam lasts 30 minutes (15 minutes with your eyes open and 15 minutes with your eyes closed - relaxed wakefulness). The analyses are performed by a certified neurophysiologist (STS).

**Safety screening**

Adverse Events Questionnaire (AEQ): Questionnaire that must be answered after each stimulation session to assess adverse effects such as tingling sensations, itching, mild transient redness of the skin and discomfort on the region of stimulation, moderate fatigue, difficulty concentrating, headache, and nauseas. (45)

**Intervention**

*Transcranial direct current stimulation (tDCS)*

Both anodal and sham tDCS will be delivered by the same battery-driven (neuroConn: DC Stimulator Plus), for 20 minutes. The research assistant will set up the device according to the assignment list in order of participant's registration number.

For peer review only

Saline-soaked surface 35cm² (5 cm X 7cm) sponge electrodes connected to the stimulator will be placed upon the patient's scalp and secured with adjustable rubber straps.

The sponge placement follows the 10-20 EEG system. Group 1 - bilateral temporal cortex (BTC) - two anode electrodes are placed over T3 and T4 respectively, and the cathode electrode over the supraorbital region (FP2). Group 2 – left dorsolateral prefrontal cortex (lDLPFC), the anode electrode is placed over F3 and the cathode over FP2. Group 3 – sham group – half of the participants are following the montage of group 1 (BTC) and the other half from group 2 (lDLPFC). For the sham stimulation, patients receive the active current with ramping up and down for 30 seconds to simulate the real stimulation, as referred by other studies. (45,46) Patients are monitored daily for side effects, according to international safety guidelines, and with the Adverse Events Questionnaire (AEQ). (47)

*Cognitive Training*

The Rehacom is a cognitive software for patients with different etiologies approved by the Brazilian Health Regulatory Agency (ANVISA). This software has several cognitive modules. For the purpose of the present study, we are using the attentional visual and verbal memory training tasks with increasing levels of difficulty according to the patient's performance. During the training, the feedback option is active, so the patient can be oriented and improve his/her performance over the trials. The initial level is adjusted to level 1 for all patients who have incomplete high school, for those who complete high school the starting level is 4, and for those with complete college, the starting level is 5. (48–50)

The cognitive training follows two possible random sequence order – memory/ attention or attention/memory modules, always alternating daily up to the end of the last

For peer review only

stimulation session. Each training has a 20-minute duration, always concomitant to the tDCS.

**Sample size calculation**

The sample calculation was performed using the software GPower 3.1, using the statistical 2-wayANOVA (3 groups and 3 timepoints), a given α 5%, power 80% and, interaction effect of 0.25 considering the primary outcome. G power analysis provided a sample size of 36 participants based on the F calculation.

**Statistical analysis**

Descriptive statistics are used to report demographic data. Kolmogorov-Smirnov test was used to test data normality. To analyze the primary outcome, changes on episodic memory (RAVLT scores), we will use ANOVA for normal data distribution or non-parametric tests. We assume that each participant has a random effect on the model. For the secondary outcome (EEG spectral power), we plan to use the mixed effect model (reml), considering group and time as fixed factors and each participant as a random effect. Estimated alpha value of 5%. An intention-to-treat framework will be applied.

**Ethics and dissemination**

TDCS is a safe intervention not only because the electric current applied is very low (0.5 - 2mA over a 25-35 $cm^2$ area), but also because the electrodes embedded in saline solution minimize tissue resistance, avoid overheating. Tingling sensations, itching, mild transient redness of the skin and discomfort on the region of stimulation, moderate fatigue, difficulty concentrating, headache and nauseas are possible adverse effects, but these effects do not usually last long and are often seen at the same frequency in experimental and placebo groups. (10,51,52) The safety side-effect questionnaire (AEQ (31)) is collected after each stimulation session. Complaints regarding the stimulation or high

AEQ scores are reported to the safety board and the medical coverage may be called for necessary care. Written informed consent for participation in the study will be obtained from all participants. Participant information is stored securely in locked file cabinets and participant digital information is password protected.

**Results**

Based on the previous studies in TBI and tDCS, we expect that (1) improvement on the memory scores in the active tDCS group compared to the sham group; (2) memory scores will be higher after the BTC active tDCS compared to the lDLPFC; (3) we hypothesize that measures of the EEG will show significant delta reduction and an increase in alpha waves close to the of active sponge placement compared to the sham group.

**DISCUSSION**

In order to contribute to the development of evidence-based rehabilitation treatments to TBI survivors with memory impairments, the present study aims to investigate whether the concomitant use of tDCS targeting the BTC or the lDLFFC concomitant to the computer-based cognitive training improves memory performance in patients with moderate and severe closed TBI patients.

Indeed, TBI causes health loss and disability for individuals and their families (53) and memory impairment is one of the most frequent cognitive complaints, (54,55) an effective rehabilitation tool will be helpful to improve this burden in this population.

There is evidence that tDCS may improve cognitive impairments, such as memory impairments, following TBI and other etiologies. (56,57) Prior research has shown the efficacy of anodal tDCS in improving memory performance during tasks such as face-name associative recall tasks, intentional memorization of words, figure-naming tests, word recall and picture-pseudoword associative learning tasks. (58–62) A recent

For peer review only

systematic review found 14 experimental studies on adult TBI patients who received tDCS for the assessment of clinical or surrogate outcomes (63) and, to our knowledge, just two studies used tDCS concomitant to cognitive training in TBI patients. (20,22)

Despite some disadvantages, namely poor spatial/temporal resolution and stimulation of large part of the brain, there are many advantages to tDCS, such as low risk of adverse effects and low cost. (64) It has been proven that tDCS does not induce depolarization, meaning it does not induce the firing of neurons when they are not near threshold. Therefore it is less likely that neurons not engaged in the task at hand will discharge, hence the importance of applying tDCS during a specific task in order to target a particular circuitry. (23) It has also been suggested that more systematic investigations are needed, due to the heterogeneity of findings in tDCS research and the different parameters used in the stimulation. (59,65)

EEG will be used to guarantee safety (32–35) and to measure cortical activity post intervention. Spikes and abnormal waves shown on the EEG will provide clinical guidance on whether to include the participant in the present study. We expect to reduce delta activities and increase alpha frequencies close to the active electrodes and find a better performance correlation in neuropsychological tests in the active group, as demonstrated previously. (24)

This is a study to test the effectiveness of combined tDCS and cognitive training to improve episodic memory in patients with TBI. The results generated may potentially be useful for other neurological disorders that cause cognitive impairments. Our open-label pilot study (n=4 participants) has proven the feasibility of the method and a moderate effect size of the RAVL scores between the baseline to the last tDCS session. Results will be presented at conferences and submitted for publication in peer-reviewed journals.

For peer review only

**Trial Status**

This trial was registered on the website clinicaltrials.gov with the registration number NCT04540783. The open-label pilot study was performed with 4 participants in 2018 and validated the study protocol. Recruitment started in February 2019. At the time of submission of this paper, we had completed 15 participants. The programmed completion date for the primary outcome is June 2022.

This study will provide important data regarding the use of the combined tools to improve the memory of persons that suffer from the sequela of a TBI. Longitudinal clinical studies are required to further interrogate the clinical efficacy of this technique to improve the mood and the quality of life of this target population.

**Acknowledgements**

The authors thank the Institute of Psychiatry, Hospital das Clínicas (IPq HCFMUSP), the secretary Sandra Aparecida de Lima Falcon and all participants and their families for their support and assistance.

**Conflicts of Interests**

Authors report no conflict of interests.

**Data**

Technical appendix, statistical code, and dataset will be available from the following link: https://drive.google.com/file/d/1WLV4yowqcyU5DuHW9PBI3FQtNrS2WKWe/view?usp=sharing

**Author Contributions**

Research project: Conception – ALZ, WSP.

Organization – ALZ, WSP, ARB, LV, VMG.

Execution – DJF, VMP, DC, STS.

Statistical Analysis: Design – ALZ, WSP, ARB

For peer review only

Manuscript Preparation: First draft – DJF. Final draft – DJF, VMP, DC, ALZ

Review and Critique: ALZ, ARB, LV, VMG, DC, DJF, VMP.

**Funding**

Vanessa M. Paglioni reports grant from Coordination for the Improvement of Higher Education Personnel (CAPES) – Brazil.

Dr. Ana Luiza Zaninotto is supported by the National Institute of Health 3R01DC017291 – 02.

Dr. Wellingson Silva Paiva is supported by the NIHR Global Health Research Group on Neurotrauma, which was commissioned by the National Institute for Health Research (NIHR) using UK aid from the UK Government (project 16/137/105).

The views expressed in this publication are those of the author(s) and not necessarily those of the NIH, NIDCD, NIHR or the Department of Health and Social Care.

**ORCID IDs**

Dagliê Jorge de Freitas https://orcid.org/0000-0002-2473-6210

Daniel de Carvalho https://orcid.org/0000-0003-4960-0800

Vanessa M. Paglioni https://orcid.org/0000-0002-8008-2231

Andre R. Brunoni https://orcid.org/0000-0002-6310-3571

Leandro Valiengo https://orcid.org/0000-0002-7628-9993

Sigride Thome-Souza https://orcid.org/0000-0002-3017-6723

Vinicius Monteiro de Paula Guirado https://orcid.org/0000-0002-7782-3609

Ana Luiza Zaninotto https://orcid.org/0000-0003-0639-0612

Wellingson S. Paiva https://orcid.org/0000-0002-5165-7040

**Legend for Figure 1:** Figure 1 CONSORT Flow Diagram

For peer review only

## REFERENCES

1 Menon DK, Schwab K, Wright DW, Maas AI. Position statement: Definition of traumatic brain injury. Vol. 91, *Arch Phys Med Rehabil* 2010;91:1637–40.
2 Gentleman SM, Roberts GW, Gennarelli TA, Maxwell WL, Adams JH, Kerr S, et al. Axonal injury: a universal consequence of fatal closed head injury? *Acta Neuropathol* 1995;89:537–43.
3 Miotto EC, Cinalli FZ, Serrao VT, Benute GG, Lucia MCS, Scaff M. Cognitive deficits in patients with mild to moderate traumatic brain injury. *Arq. Neuro-Psiquiatr* 2010;68:862–8.
4 Wright MJ, Schmitter-Edgecombe M. The impact of verbal memory encoding and consolidation deficits during recovery from moderate-to-severe traumatic brain injury. *J Head Trauma Rehabil* 2011;26:182–91.
5 Vakil E, Greenstein Y, Weiss I, Shtein S. The Effects of Moderate-to-Severe Traumatic Brain Injury on Episodic Memory: a Meta-Analysis. *Neuropsychol Rev* 2019;29:270–87.
6 Moscovitch M, Cabeza R, Winocur G, Nadel L. Episodic Memory and Beyond: The Hippocampus and Neocortex in Transformation. *Annu Rev Psychol* 2016;67:105–34.
7 Eichenbaum H. Prefrontal-hippocampal interactions in episodic memory. *Nat Rev Neurosci* 2017;18:547–58.
8 Ponsford JL, Downing MG, Olver J, Ponsford M, Acher R, Carty M, et al. Longitudinal follow-up of patients with traumatic brain injury: Outcome at two, five, and ten years post-injury. *J Neurotrauma* 2014;31:64–77.
9 Nitsche MA, Liebetanz D, Lang N, Antal A, Tergau F, Paulus W. Safety criteria for transcranial direct current stimulation (tDCS) in humans. *Clin Neurophysiol* 2003;114:2220–2.
10 Nitsche MA, Cohen LG, Wassermann EM, Priori A, Lang N, Antal A, et al. Transcranial direct current stimulation: State of the art 2008. *Brain Stimul* 2008;1:206–23.
11 Levin HS. Memory deficit after closed-head injury. *J Clin Exp Neuropsychol* 1990;12:129–53.
12 Li S, Zaninotto AL, Neville IS, Paiva WS, Nunn D, Fregni F. Clinical utility of brain stimulation modalities following traumatic brain injury: Current evidence. *Neuropsychiatr Dis Treat* 2015;11:1573–86.
13 Amatachaya A, Auvichayapat N, Patjanasoontorn N, Suphakunpinyo C, Ngernyam N, Aree-uea B, et al. Effect of Anodal Transcranial Direct Current Stimulation on Autism: A Randomized Double-Blind Crossover Trial. *Behav Neurol* 2014;2014:1–7.
14 Palacios EM, Sala-Llonch R, Junque C, Fernandez-Espejo D, Roig T, Tormos JM, et al. Long-term declarative memory deficits in diffuse TBI: Correlations with cortical thickness, white matter integrity and hippocampal volume. *Cortex* 2013;49:646–57.
15 Sozda CN, Muir JJ, Springer US, Partovi D, Cole MA. Differential learning and memory performance in OEF/OIF veterans for verbal and visual material. *Neuropsychology* 2014;28:347–52.
16 Zaninotto AL, Vicentini JE, Solla DJF, Silva TT, De Paula Guirado VM, Feltrin F, et al. Visuospatial memory improvement in patients with diffuse axonal injury (DAI): A 1-year follow-up study. *Acta Neuropsychiatr* 2017;29:35–42.
17 Estraneo A, Pascarella A, Moretta P, Masotta O, Fiorenza S, Chirico G, et al.

For peer review only

Repeated transcranial direct current stimulation in prolonged disorders of consciousness: A double-blind cross-over study. *J Neurol Sci* 2017;375:464–70.
18 Wilke S, List J, Mekle R, Lindenberg R, Bukowski M, Ott S, et al. No effect of anodal tDCS on GABA levels in patients with recurrent mild traumatic brain injury. *J Neurotrauma* 2016;1–50.
19 Kang EK, Kim DY, Paik NJ. Transcranial direct curent stimulation of the left prefrontal cortex improves attention in patients with traumatic brain injury: A pilot study. *J Rehabil Med* 2012;44:346–50.
20 Leśniak M, Polanowska K, Seniów J, Członkowska A. Effects of repeated anodal tDCS coupled with cognitive training for patients with severe traumatic brain injury: A pilot randomized controlled trial. *J Head Trauma Rehabil* 2014;29: E20-E29.
21 Galetto V, Sacco K. Neuroplastic Changes Induced by Cognitive Rehabilitation in Traumatic Brain Injury: A Review. *Neurorehabil Neural Repair* 2017;31:800–13.
22 Sacco K, Galetto V, Dimitri D, Geda E, Perotti F, Zettin M, et al. Concomitant use of transcranial direct current stimulation and computer-assisted training for the rehabilitation of attention in traumatic brain injured patients: Behavioral and neuroimaging results. *Front Behav Neurosci* 2016;10:1–11.
23 Miniussi C, Harris JA, Ruzzoli M. Modelling non-invasive brain stimulation in cognitive neuroscience. *Neurosci Biobehav Rev* 2013;37:1702–12.
24 Ulam F, Shelton C, Richards L, Davis L, Hunter B, Fregni F, et al. Cumulative effects of transcranial direct current stimulation on EEG oscillations and attention/working memory during subacute neurorehabilitation of traumatic brain injury. *Clin Neurophysiol* 2015;126:486–96.
25 Keeser D, Padberg F, Reisinger E, Pogarell O, Kirsch V, Palm U, et al. Prefrontal direct current stimulation modulates resting EEG and event-related potentials in healthy subjects: A standardized low resolution tomography (sLORETA) study. *Neuroimage* 2010;55:644–57.
26 Balconi M, Vitaloni S. The tDCS effect on alpha brain oscillation for correct vs. incorrect object use. The contribution of the left DLPFC. *Neurosci Lett* 2012;517:25–9.
27 Maeoka H, Matsuo A, Hiyamizu M, Morioka S, Ando H. Influence of transcranial direct current stimulation of the dorsolateral prefrontal cortex on pain related emotions: A study using electroencephalographic power spectrum analysis. *Neurosci Lett* 2012;512:12–6.
28 Jacobson L, Ezra A, Berger U, Lavidor M. Modulating oscillatory brain activity correlates of behavioral inhibition using transcranial direct current stimulation. *Clin Neurophysiol* 2012;123:979–84.
29 Miller J, Berger B, Sauseng P. Anodal transcranial direct current stimulation (tDCS) increases frontal-midline theta activity in the human EEG: A preliminary investigation of non-invasive stimulation. *Neurosci Lett* 2015;588:114–9.
30 St. Louis EK, Frey LC, eds. Electroencephalography (EEG): An Introductory Text and Atlas of Normal and Abnormal Findings in Adults, Children, and Infants. Chicago, IL: American Epilepsy Society 2016.
31 Hughes JR. EEG in clinical practice. Newton, MA: Butterworth-Heinemann 1994:242
32 Liu A, Bryant A, Jefferson A, Friedman D, Minhas P, Barnard S, et al. Exploring the efficacy of a 5-day course of transcranial direct current stimulation (TDCS) on depression and memory function in patients with well-controlled temporal

For peer review only

lobe epilepsy. *Epilepsy Behav* 2016;55:11–20.
33 Antal A, Boros K, Poreisz C, Chaieb L, Terney D, Paulus W. Comparatively weak after-effects of transcranial alternating current stimulation (tACS) on cortical excitability in humans. *Brain Stimul* 2008;1:97–105.
34 Zaehle T, Sandmann P, Thorne JD, Jäncke L, Herrmann CS. Transcranial direct current stimulation of the prefrontal cortex modulates working memory performance: Combined behavioural and electrophysiological evidence. *BMC Neurosci* 2011;12:9–14.
35 Tadini L, El-Nazer R, Brunoni AR, Williams J, Carvas M, Boggio P, et al. Cognitive, mood, and electroencephalographic effects of noninvasive cortical stimulation with weak electrical currents. *J ECT* 2011;27:134–40.
36 Hannawi Y, Stevens RD. Mapping the connectome following traumatic brain injury. *Curr Neurol Neurosci Rep* 2016;16(5).
37 Marceglia S, Mrakic-Sposta S, Rosa M, Ferrucci R, Mameli F, Vergari M, et al. Transcranial direct current stimulation modulates cortical neuronal activity in Alzheimer's disease. *Front Neurosci* 2016;10:1–11.
38 Roche RAP, Dockree PM, Garavan H, Foxe JJ, Robertson IH, O'Mara SM. EEG alpha power changes reflect response inhibition deficits after traumatic brain injury (TBI) in humans. *Neurosci Lett* 2004;362:1–5.
39 Dressler O, Schneider G, Stockmanns G, Kochs EF. Awareness and the EEG power spectrum: Analysis of frequencies. *Br J Anaesth* 2004;93:806–9.
40 Gorenstein C, Wang YP, Argimon I de L, Werlang BSG. Manual do Inventário de depressão de Beck-BDI-II. São Paulo, SP: Casa do Psicólogo 2011:172
41 Cunha JA. Manual da versão em português das Escalas Beck. São Paulo, SP: Casa do Psicólogo 2001:171
42 Denney DA, Ringe WK, Lacritz LH. Dyadic short forms of the Wechsler Adult Intelligence Scale-IV. *Arch Clin Neuropsychol* 2015;30:404–12.
43 Wechsler D. WAIS-III: Escala de inteligência Wechsler para adultos: manual técnico. São Paulo, SP: Casa do Psicólogo 2004:276
44 De Paula J, Fernandes Malloy-Diniz L. Teste de Aprendizagem Auditivo-Verbal de Rey (RAVLT). São Paulo:Vetor Editora Psico-Pedagógica Ltda. 2018.
45 Bikson M, Grossman P, Thomas C, Zannou AL, Jiang J, Adnan T, et al. Safety of Transcranial Direct Current Stimulation: Evidence Based Update 2016. *Brain Stimul* 2016;9:641–61.
46 Antal A, Alekseichuk I, Bikson M, Brockmöller J, Brunoni AR, Chen R, et al. Low intensity transcranial electric stimulation: Safety, ethical, legal regulatory and application guidelines. *Clin Neurophysiol* 2017;128:1774–809.
47 Brunoni AR, Amadera J, Berbel B, Volz MS, Rizzerio BG, Fregni F. A systematic review on reporting and assessment of adverse effects associated with transcranial direct current stimulation. *Int J Neuropsychopharmacol* 2011;14:1133–45.
48 Fernandez E, Bergado Rosado JA, Perez DR, Santana SS, Aguilar MT, Bringas ML. Effectiveness of a computer-based training program of attention and memory in patients with acquired brain damage. *Behav Sci* 2018;8.
49 Yoo C, Yong MH, Chung J, Yang Y. Effect of computerized cognitive rehabilitation program on cognitive function and activities of living in stroke patients. *J Phys Ther Sci* 2015;27:2487–9.
50 Veisi-Pirkoohi S, Hassani-Abharian P, Kazemi R, Vaseghi S, Zarrindast MR, Nasehi M. Efficacy of RehaCom cognitive rehabilitation software in activities of daily living, attention and response control in chronic stroke patients. *J Clin*

*Neurosci* 2020;71:101–7.
51 Brunoni A, Nitsche M, Bolognini N, Bikson M, Wagner T, Merabet L, et al. Clinical research with tDCS: Challenges and future directions. *Brain Stimul* 2012;5:175–95.
52 de Amorim RLO, Brunoni AR, de Oliveira MAF, Zaninotto ALC, Nagumo MM, Guirado VM de P, et al. Transcranial direct current stimulation for post-concussion syndrome: Study protocol for a randomized crossover trial. *Front Neurol* 2017 May 2;8:164.
53 James SL, Theadom A, Ellenbogen RG, Bannick MS, Montjoy-Venning W, Lucchesi LR, et al. Global, regional, and national burden of traumatic brain injury and spinal cord injury, 1990–2016: a systematic analysis for the Global Burden of Disease Study 2016. *Lancet Neurol* 2019 Jan 1 [cited 2020 Jun 26];18:56–87.
54 Jourdan C, Bayen E, Pradat-Diehl P, Ghout I, Darnoux E, Azerad S, et al. A comprehensive picture of 4-year outcome of severe brain injuries. Results from the PariS-TBI study. *Ann Phys Rehabil Med* 2016;59:100–6.
55 Zec RF, Zellers D, Belman J, Miller J, Matthews J, Ferneau-Belman D, et al. Long-term consequences of severe closed head injury on episodic memory. *J Clin Exp Neuropsychol* 2001;23:671–91.
56 Sandrini M, Brambilla M, Manenti R, Rosini S, Cohen LG, Cotelli M. Noninvasive stimulation of prefrontal cortex strengthens existing episodic memories and reduces forgetting in the elderly. *Front Aging Neurosci* 2014;6:1–9.
57 Park SH, Koh EJ, Choi HY, Ko MH. A double-blind, sham-controlled, pilot study to assess the effects of the concomitant use of transcranial direct current stimulation with the computer assisted cognitive rehabilitation to the prefrontal cortex on cognitive functions in patients with stroke. *J Korean Neurosurg Soc* 2013;54(6):484–8.
58 Leshikar ED, Leach RC, McCurdy MP, Trumbo MC, Sklenar AM, Frankenstein AN, et al. Transcranial direct current stimulation of dorsolateral prefrontal cortex during encoding improves recall but not recognition memory. *Neuropsychologia* 2017;106:390–7.
59 Medvedeva A, Materassi M, Neacsu V, Beresford-Webb J, Hussin A, Khan N, et al. Effects of Anodal Transcranial Direct Current Stimulation Over the Ventrolateral Prefrontal Cortex on Episodic Memory Formation and Retrieval. *Cereb Cortex* 2019;29:657–65.
60 Fileccia E, Di Stasi V, Poda R, Rizzo G, Stanzani-Maserati M, Oppi F, et al. Effects on cognition of 20-day anodal transcranial direct current stimulation over the left dorsolateral prefrontal cortex in patients affected by mild cognitive impairment: a case-control study. *Neurol Sci* 2019;40:1865–72.
61 Sandrini M, Manenti R, Gobbi E, Rusich D, Bartl G, Cotelli M. Transcranial direct current stimulation applied after encoding facilitates episodic memory consolidation in older adults. *Neurobiol Learn Mem* 2019;163:107037.
62 Antonenko D, Thams F, Uhrich J, Dix A, Thurm F, Li SC, et al. Effects of a multi-session cognitive training combined with brain stimulation (TRAINSTIM-COG) on age-associated cognitive decline – Study protocol for a randomized controlled phase IIb (MONOCENTER) trial. *Front Aging Neurosci* 2019;10:1–12.
63 Zaninotto AL, El-Hagrassy MM, Green JR, Babo M, Paglioni VM, Guerra Benute G, et al. Transcranial direct current stimulation (tDCS) effects on

For peer review only

traumatic brain injury (TBI) recovery A systematic review. *Dement Neuropsychol* 2019 [cited 2019 Jul 2];13:172–9.

64 Miniussi C, Ruzzoli M. Transcranial stimulation and cognition. In: Lozano AM, Hallett M, eds. Handbook of Clinical Neurology Elsevier B.V. 2013: 739–50.

65 Galli G, Vadillo MA, Sirota M, Feurra M, Medvedeva A. A systematic review and meta-analysis of the effects of transcranial direct current stimulation (tDCS) on episodic memory. *Brain Stimul* 2019;12:231–41.

For peer review only

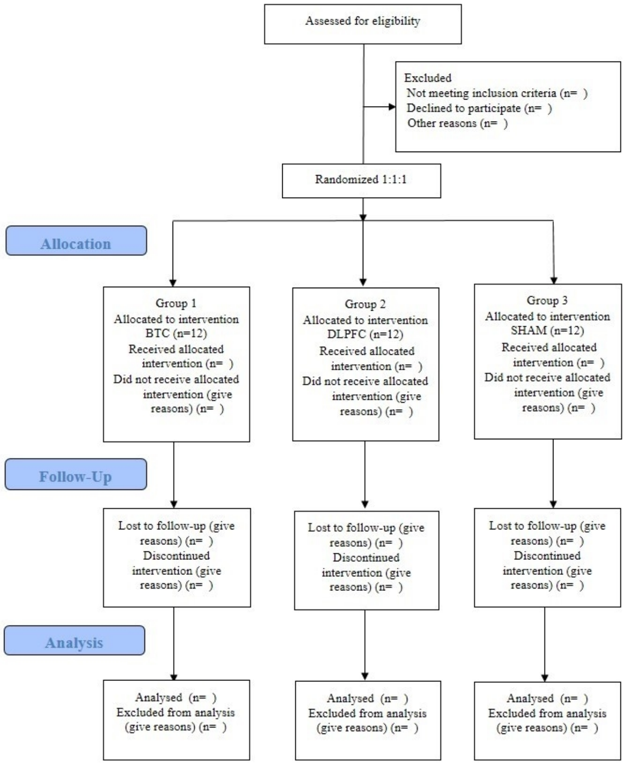


Figure 1 CONSORT Flow Diagram

190x338mm (96 x 96 DPI)

# CONSORT 2010 checklist of information to include when reporting a randomised trial*

| Section/Topic | Item No | Checklist item: The effects of transcranial direct current stimulation (tDCS) and cognitive training on episodic memory in patients with traumatic brain injury: a double-blind, randomized, placebo-controlled study | Reported on page No |
|---|---|---|---|
| **Title and abstract** | | | |
| | 1a | Identification as a randomised trial in the title | 1 |
| | 1b | Structured summary of trial design, methods, results, and conclusions (for specific guidance see CONSORT for abstracts) | 2 |
| **Introduction** | | | |
| Background and objectives | 2a | Scientific background and explanation of rationale | 4-6 |
| | 2b | Specific objectives or hypotheses | 6 |
| **Methods** | | | |
| Trial design | 3a | Description of trial design (such as parallel, factorial) including allocation ratio | 6 |
| | 3b | Important changes to methods after trial commencement (such as eligibility criteria), with reasons | n/a |
| Participants | 4a | Eligibility criteria for participants | 8 |
| | 4b | Settings and locations where the data were collected | 6 |
| Interventions | 5 | The interventions for each group with sufficient details to allow replication, including how and when they were actually administered | 10-11 |
| Outcomes | 6a | Completely defined pre-specified primary and secondary outcome measures, including how and when they were assessed | 9-10 |
| | 6b | Any changes to trial outcomes after the trial commenced, with reasons | n/a |
| Sample size | 7a | How sample size was determined | 11-12 |
| | 7b | When applicable, explanation of any interim analyses and stopping guidelines | n/a |
| Randomisation: | | | |
| Sequence generation | 8a | Method used to generate the random allocation sequence | 7 |
| | 8b | Type of randomisation; details of any restriction (such as blocking and block size) | 7 |
| Allocation concealment mechanism | 9 | Mechanism used to implement the random allocation sequence (such as sequentially numbered containers), describing any steps taken to conceal the sequence until interventions were assigned | 7 |
| Implementation | 10 | Who generated the random allocation sequence, who enrolled participants, and who assigned participants to interventions | 7 |

For peer review only



For peer review only - http://bmjopen.bmj.com/site/about/guidelines.xhtml

| | | | |
|---|---|---|---|
| Blinding | 11a | If done, who was blinded after assignment to interventions (for example, participants, care providers, those assessing outcomes) and how | 7 |
| | 11b | If relevant, description of the similarity of interventions | 7 |
| Statistical methods | 12a | Statistical methods used to compare groups for primary and secondary outcomes | 12 |
| | 12b | Methods for additional analyses, such as subgroup analyses and adjusted analyses | n/a |
| **Results** | | | |
| Participant flow (a diagram is strongly recommended) | 13a | For each group, the numbers of participants who were randomly assigned, received intended treatment, and were analysed for the primary outcome | n/a |
| | 13b | For each group, losses and exclusions after randomisation, together with reasons | n/a |
| Recruitment | 14a | Dates defining the periods of recruitment and follow-up | n/a |
| | 14b | Why the trial ended or was stopped | n/a |
| Baseline data | 15 | A table showing baseline demographic and clinical characteristics for each group | n/a |
| Numbers analysed | 16 | For each group, number of participants (denominator) included in each analysis and whether the analysis was by original assigned groups | n/a |
| Outcomes and estimation | 17a | For each primary and secondary outcome, results for each group, and the estimated effect size and its precision (such as 95% confidence interval) | n/a |
| | 17b | For binary outcomes, presentation of both absolute and relative effect sizes is recommended | n/a |
| Ancillary analyses | 18 | Results of any other analyses performed, including subgroup analyses and adjusted analyses, distinguishing pre-specified from exploratory | n/a |
| Harms | 19 | All important harms or unintended effects in each group (for specific guidance see CONSORT for harms) | n/a |
| **Discussion** | | | |
| Limitations | 20 | Trial limitations, addressing sources of potential bias, imprecision, and, if relevant, multiplicity of analyses | 14 |
| Generalisability | 21 | Generalisability (external validity, applicability) of the trial findings | 14 |
| Interpretation | 22 | Interpretation consistent with results, balancing benefits and harms, and considering other relevant evidence | n/a |
| **Other information** | | | |
| Registration | 23 | Registration number and name of trial registry | 15 |
| Protocol | 24 | Where the full trial protocol can be accessed, if available | 15 |
| Funding | 25 | Sources of funding and other support (such as supply of drugs), role of funders | 16 |

*We strongly recommend reading this statement in conjunction with the CONSORT 2010 Explanation and Elaboration for important clarifications on all the items. If relevant, we also recommend reading CONSORT extensions for cluster randomised trials, non-inferiority and equivalence trials, non-pharmacological treatments, herbal interventions, and pragmatic trials. Additional extensions are forthcoming: for those and for up to date references relevant to this checklist, see www.consort-statement.org.


For peer review only - http://bmjopen.bmj.com/site/about/guidelines.xhtml

# Reporting checklist for protocol of a clinical trial.

Based on the SPIRIT guidelines.

## Instructions to authors

Complete this checklist by entering the page numbers from your manuscript where readers will find each of the items listed below.

Your article may not currently address all the items on the checklist. Please modify your text to include the missing information. If you are certain that an item does not apply, please write "n/a" and provide a short explanation.

Upload your completed checklist as an extra file when you submit to a journal.

In your methods section, say that you used the SPIRITreporting guidelines, and cite them as:

Chan A-W, Tetzlaff JM, Altman DG, Laupacis A, Gøtzsche PC, Krleža-Jerić K, Hróbjartsson A, Mann H, Dickersin K, Berlin J, Doré C, Parulekar W, Summerskill W, Groves T, Schulz K, Sox H, Rockhold FW, Rennie D, Moher D. SPIRIT 2013 Statement: Defining standard protocol items for clinical trials. Ann Intern Med. 2013;158(3):200-207

| | | Reporting Item | Page Number |
|---|---|---|---|
| **Administrative information** | | | |
| Title | #1 | Descriptive title identifying the study design, population, interventions, and, if applicable, trial acronym | 1 |

For peer review only

| | | | |
|---|---|---|---|
| Trial registration | #2a | Trial identifier and registry name. If not yet registered, name of intended registry | 2 |
| Trial registration: data set | #2b | All items from the World Health Organization Trial Registration Data Set | 14 |
| Protocol version | #3 | Date and version identifier | n/a |
| Funding | #4 | Sources and types of financial, material, and other support | 15 |
| Roles and responsibilities: contributorship | #5a | Names, affiliations, and roles of protocol contributors | 15 |
| Roles and responsibilities: sponsor contact information | #5b | Name and contact information for the trial sponsor | n/a |
| Roles and responsibilities: sponsor and funder | #5c | Role of study sponsor and funders, if any, in study design; collection, management, analysis, and interpretation of data; writing of the report; and the decision to submit the report for publication, including whether they will have ultimate authority over any of these activities | 15 |
| Roles and responsibilities: committees | #5d | Composition, roles, and responsibilities of the coordinating centre, steering committee, endpoint adjudication committee, data management team, and other individuals or groups overseeing the trial, if applicable (see Item 21a for data monitoring committee) | 7 |

For peer review only

**Introduction**

| | | | |
|---|---|---|---|
| Background and rationale | #6a | Description of research question and justification for undertaking the trial, including summary of relevant studies (published and unpublished) examining benefits and harms for each intervention | 4-6 |
| Background and rationale: choice of comparators | #6b | Explanation for choice of comparators | 4-6 |
| Objectives | #7 | Specific objectives or hypotheses | 6 |
| Trial design | #8 | Description of trial design including type of trial (eg, parallel group, crossover, factorial, single group), allocation ratio, and framework (eg, superiority, equivalence, non-inferiority, exploratory) | 6 |

**Methods: Participants, interventions, and outcomes**

| | | | |
|---|---|---|---|
| Study setting | #9 | Description of study settings (eg, community clinic, academic hospital) and list of countries where data will be collected. Reference to where list of study sites can be obtained | 7 |
| Eligibility criteria | #10 | Inclusion and exclusion criteria for participants. If applicable, eligibility criteria for study centres and | 7-8 |

For peer review only

| | | | |
|---|---|---|---|
| | | individuals who will perform the interventions (eg, surgeons, psychotherapists) | |
| Interventions: description | #11a | Interventions for each group with sufficient detail to allow replication, including how and when they will be administered | 10-11 |
| Interventions: modifications | #11b | Criteria for discontinuing or modifying allocated interventions for a given trial participant (eg, drug dose change in response to harms, participant request, or improving / worsening disease) | n/a |
| Interventions: adherance | #11c | Strategies to improve adherence to intervention protocols, and any procedures for monitoring adherence (eg, drug tablet return; laboratory tests) | n/a |
| Interventions: concomitant care | #11d | Relevant concomitant care and interventions that are permitted or prohibited during the trial | n/a |
| Outcomes | #12 | Primary, secondary, and other outcomes, including the specific measurement variable (eg, systolic blood pressure), analysis metric (eg, change from baseline, final value, time to event), method of aggregation (eg, median, proportion), and time point for each outcome. Explanation of the clinical relevance of chosen efficacy and harm outcomes is strongly recommended | 9 |
| Participant timeline | #13 | Time schedule of enrolment, interventions (including any run-ins and washouts), assessments, and visits for participants. A schematic diagram is highly recommended | 8 |

For peer review only

| | | | |
|---|---|---|---|
| | | (see Figure) | |
| Sample size | #14 | Estimated number of participants needed to achieve study objectives and how it was determined, including clinical and statistical assumptions supporting any sample size calculations | 11 |
| Recruitment | #15 | Strategies for achieving adequate participant enrolment to reach target sample size | 7 |
| **Methods: Assignment of interventions (for controlled trials)** | | | |
| Allocation: sequence generation | #16a | Method of generating the allocation sequence (eg, computer-generated random numbers), and list of any factors for stratification. To reduce predictability of a random sequence, details of any planned restriction (eg, blocking) should be provided in a separate document that is unavailable to those who enrol participants or assign interventions | 7 |
| Allocation concealment mechanism | #16b | Mechanism of implementing the allocation sequence (eg, central telephone; sequentially numbered, opaque, sealed envelopes), describing any steps to conceal the sequence until interventions are assigned | 7 |
| Allocation: implementation | #16c | Who will generate the allocation sequence, who will enrol participants, and who will assign participants to interventions | 7 |

For peer review only

| | | | |
|---|---|---|---|
| Blinding (masking) | #17a | Who will be blinded after assignment to interventions (eg, trial participants, care providers, outcome assessors, data analysts), and how | 7 |
| Blinding (masking): emergency unblinding | #17b | If blinded, circumstances under which unblinding is permissible, and procedure for revealing a participant's allocated intervention during the trial | 7 |
| **Methods: Data collection, management, and analysis** | | | |
| Data collection plan | #18a | Plans for assessment and collection of outcome, baseline, and other trial data, including any related processes to promote data quality (eg, duplicate measurements, training of assessors) and a description of study instruments (eg, questionnaires, laboratory tests) along with their reliability and validity, if known. Reference to where data collection forms can be found, if not in the protocol | 9 |
| Data collection plan: retention | #18b | Plans to promote participant retention and complete follow-up, including list of any outcome data to be collected for participants who discontinue or deviate from intervention protocols | n/a |
| Data management | #19 | Plans for data entry, coding, security, and storage, including any related processes to promote data quality (eg, double data entry; range checks for data values). | 12 |

For peer review only

| | | | |
|---|---|---|---|
| | | Reference to where details of data management procedures can be found, if not in the protocol | |
| Statistics: outcomes | #20a | Statistical methods for analysing primary and secondary outcomes. Reference to where other details of the statistical analysis plan can be found, if not in the protocol | 11 |
| Statistics: additional analyses | #20b | Methods for any additional analyses (eg, subgroup and adjusted analyses) | 11 |
| Statistics: analysis population and missing data | #20c | Definition of analysis population relating to protocol non-adherence (eg, as randomised analysis), and any statistical methods to handle missing data (eg, multiple imputation) | 11 |
| **Methods: Monitoring** | | | |
| Data monitoring: formal committee | #21a | Composition of data monitoring committee (DMC); summary of its role and reporting structure; statement of whether it is independent from the sponsor and competing interests; and reference to where further details about its charter can be found, if not in the protocol. Alternatively, an explanation of why a DMC is not needed | 7 |
| Data monitoring: interim analysis | #21b | Description of any interim analyses and stopping guidelines, including who will have access to these interim results and make the final decision to terminate the trial | n/a |
| Harms | #22 | Plans for collecting, assessing, reporting, and managing solicited and spontaneously reported adverse events and other unintended effects of trial interventions or trial | 12 |

For peer review only

| | | | |
|---|---|---|---|
| | | conduct | |
| Auditing | #23 | Frequency and procedures for auditing trial conduct, if any, and whether the process will be independent from investigators and the sponsor | 7 |
| **Ethics and dissemination** | | | |
| Research ethics approval | #24 | Plans for seeking research ethics committee / institutional review board (REC / IRB) approval | 7 |
| Protocol amendments | #25 | Plans for communicating important protocol modifications (eg, changes to eligibility criteria, outcomes, analyses) to relevant parties (eg, investigators, REC / IRBs, trial participants, trial registries, journals, regulators) | n/a |
| Consent or assent | #26a | Who will obtain informed consent or assent from potential trial participants or authorised surrogates, and how (see Item 32) | 15 |
| Consent or assent: ancillary studies | #26b | Additional consent provisions for collection and use of participant data and biological specimens in ancillary studies, if applicable | n/a |
| Confidentiality | #27 | How personal information about potential and enrolled participants will be collected, shared, and maintained in order to protect confidentiality before, during, and after the trial | 12 |
| Declaration of | #28 | Financial and other competing interests for principal | 15 |

For peer review only

| | | | |
|---|---|---|---|
| interests | | investigators for the overall trial and each study site | |
| Data access | #29 | Statement of who will have access to the final trial dataset, and disclosure of contractual agreements that limit such access for investigators | 15 |
| Ancillary and post trial care | #30 | Provisions, if any, for ancillary and post-trial care, and for compensation to those who suffer harm from trial participation | n/a |
| Dissemination policy: trial results | #31a | Plans for investigators and sponsor to communicate trial results to participants, healthcare professionals, the public, and other relevant groups (eg, via publication, reporting in results databases, or other data sharing arrangements), including any publication restrictions | 14 |
| Dissemination policy: authorship | #31b | Authorship eligibility guidelines and any intended use of professional writers | n/a |
| Dissemination policy: reproducible research | #31c | Plans, if any, for granting public access to the full protocol, participant-level dataset, and statistical code | 15 |
| **Appendices** | | | |
| Informed consent materials | #32 | Model consent form and other related documentation given to participants and authorised surrogates | n/a |
| Biological specimens | #33 | Plans for collection, laboratory evaluation, and storage of biological specimens for genetic or molecular analysis in the current trial and for future use in ancillary studies, if applicable | n/a |

For peer review only

None The SPIRIT checklist is distributed under the terms of the Creative Commons Attribution License CC-BY-ND 3.0. This checklist can be completed online using https://www.goodreports.org/, a tool made by the EQUATOR Network in collaboration with Penelope.ai

For peer review only

# The effects of transcranial direct current stimulation (tDCS) and concurrent cognitive training on episodic memory in patients with traumatic brain injury: a double-blind, randomized, placebo-controlled study

| | |
|---|---|
| Journal: | *BMJ Open* |
| Manuscript ID | bmjopen-2020-045285.R1 |
| Article Type: | Protocol |
| Date Submitted by the Author: | 13-Mar-2021 |
| Complete List of Authors: | De Freitas, Daglie; HCFMUSP, Neurology/Neurosurgery<br>De Carvalho, Daniel; HCFMUSP, Neurology/Neurosurgery<br>Paglioni, Vanessa Maria; HCFMUSP, Neurology/Neurosurgery<br>Brunoni, Andre; University of São Paulo, Division of Psychiatry and Psycology<br>Valiengo, Leandro; University of Sao Paulo, Interdisciplinary Center for Applied Neuromodulation and Service of Interdisciplinary Neuromodulation<br>Souza, Maria Sigride; University of Sao Paulo, Psychiatry Department<br>Guirado, Vinícius; HCFMUSP, Division of Neurosurgery<br>Zaninotto, Ana Luiza; MGH Institute of Health Professions, Speech and Feeding Disorders Lab<br>Paiva, Wellingson; University of São Paulo, Division of Neurosurgery |
| <b>Primary Subject Heading</b>: | Neurology |
| Secondary Subject Heading: | Rehabilitation medicine |
| Keywords: | Neurological injury < NEUROLOGY, Rehabilitation medicine < INTERNAL MEDICINE, Clinical trials < THERAPEUTICS |
| | |

SCHOLARONE™
Manuscripts

*I, the Submitting Author has the right to grant and does grant on behalf of all authors of the Work (as defined in the below author licence), an exclusive licence and/or a non-exclusive licence for contributions from authors who are: i) UK Crown employees; ii) where BMJ has agreed a CC-BY licence shall apply, and/or iii) in accordance with the terms applicable for US Federal Government officers or employees acting as part of their official duties; on a worldwide, perpetual, irrevocable, royalty-free basis to BMJ Publishing Group Ltd ("BMJ") its licensees and where the relevant Journal is co-owned by BMJ to the co-owners of the Journal, to publish the Work in this journal and any other BMJ products and to exploit all rights, as set out in our licence.*

*The Submitting Author accepts and understands that any supply made under these terms is made by BMJ to the Submitting Author unless you are acting as an employee on behalf of your employer or a postgraduate student of an affiliated institution which is paying any applicable article publishing charge ("APC") for Open Access articles. Where the Submitting Author wishes to make the Work available on an Open Access basis (and intends to pay the relevant APC), the terms of reuse of such Open Access shall be governed by a Creative Commons licence – details of these licences and which Creative Commons licence will apply to this Work are set out in our licence referred to above.*

*Other than as permitted in any relevant BMJ Author's Self Archiving Policies, I confirm this Work has not been accepted for publication elsewhere, is not being considered for publication elsewhere and does not duplicate material already published. I confirm all authors consent to publication of this Work and authorise the granting of this licence.*

For peer review only

# The effects of transcranial direct current stimulation (tDCS) and concurrent cognitive training on episodic memory in patients with traumatic brain injury: a double-blind, randomized, placebo-controlled study

Dagliê Jorge de Freitas [1], Daniel de Carvalho[1], Vanessa M. Paglioni[1], Andre R. Brunoni[2,3], Leandro Valiengo[2,3], Sigride Thome-Souza[4], Vinicius Monteiro de Paula Guirado[1], Ana Luiza Zaninotto[1,5]*, Wellingson S. Paiva[1]*

*Sharing the last authorship.

1. Department of Neurology/ Neurosurgery, Clinics Hospital, Medical School of University of Sao Paulo (HC-FMUSP), Brazil.
2. Interdisciplinary Center for Applied Neuromodulation and Service of Interdisciplinary Neuromodulation, LIM-27, University of São Paulo, Brazil
3. Departments of Internal Medicine and Psychiatry, Faculdade de Medicina da Universidade de São Paulo, Brazil
4. Service of video-electroencephalography, Psychiatry Department, School of Medicine, University of Sao Paulo, Sao Paulo, Brazil.
5. Speech and Feeding Disorders Lab, MGH Institute of Health Professions (MGHIHP), Boston, USA.

Word count: 3163

Corresponding author:
Ana Luiza Zaninotto
79/96 13th street, 2nd floor.
Boston, MA, United States
02129
Correspondence: acostazaninotto@mghihp.edu

For peer review only

ABSTRACT

**Introduction:** Deficits in episodic memory following traumatic brain injury (TBI) are common and affect independence in activities of daily living. Transcranial direct current stimulation (tDCS) and concurrent cognitive outcomes may contribute to improve episodic memory in patients with TBI. Although previous studies have shown the potential benefits of tDCS to improve cognition, the benefits of the tDCS applied simultaneously to cognitive training are inconsistent. This study aims to (1) investigate whether active tDCS combined with computer-assisted cognitive training enhances episodic memory compared to sham tDCS; (2) to compare the differences between active tDCS applied over the left dorsolateral prefrontal cortex (lDLPFC) and bilateral temporal cortex (BTC) on episodic memory; and (3) investigate inter and intra-group changes on cortical activity measured by quantitative electroencephalogram (qEEG).

**Methods and analysis:** A randomized, parallel-group, double-blind placebo-controlled study is conducted. Thirty-six participants with chronic, moderate, and severe closed TBI are being recruited and randomized into three parallel groups (1:1:1) based on the placement of tDCS sponges and activation (active or sham). TDCS is applied for 10 consecutive days for 20 minutes, combined with a computer-based cognitive training. Cognitive scores and qEEG are collected at baseline, on the last day of the stimulation session, and 3 months after the last tDCS session. We hypothesize that (1) memory scores in the active tDCS group will improve compared to the sham group; (2) BTC and lDLPFC active tDCS memory scores might be significantly different; (3) there will be significant delta reduction and an increase in alpha waves close to the location of the active sponge placement compared to the sham group.

**Ethics and dissemination:** This study was approved by Hospital das Clínicas, University of São Paulo Ethical Institutional Review Border (CAAE: 87954518.0.0000.0068)

**Trial registration number:** ClinicalTrials.gov(NCT04540783).

**Keywords:** traumatic brain injury, episodic memory, transcranial direct current stimulation, cognition, rehabilitation.

For peer review only

## ARTICLE SUMMARY

### Strengths and limitations of this study

- To our knowledge, this protocol is the first randomized controlled trial investigating the effects of the transcranial direct current stimulation (tDCS) and concurrent computer-assisted cognitive training on episodic memory in individuals with TBI.
- This study may contribute to the development of evidence-based low-risk and low-cost rehabilitation treatments for TBI survivors with memory impairments.
- This protocol will investigate how the anodal stimulation of temporal cortex compares to the stimulation of the left dorsolateral prefrontal cortex in terms of episodic memory performance.
- Electroencephalogram (EEG) will be used to evaluate changes in cortical activity after the tDCS intervention.
- Due to sample size restrictions, sex and TBI severity will not be considered as covariates, which might be a limitation of this study.

For peer review only

## INTRODUCTION

Traumatic Brain Injury (TBI) is an alteration in brain function caused by an external force and a major cause of death and disability throughout the world. (1,2) The hippocampus and the prefrontal cortex are among the brain structures more susceptible to lesions after a brain insult and, as a consequence, head injury survivors may experience difficulties in recalling specific events from the personal past and imagining novel scenarios. (3–5) Those regions are known to play important roles in episodic memory, which is a declarative memory containing information about place and time of acquisition as opposed to semantic memory, which refers to memory not tied to the context of encoding. (6) The hippocampus specifically organizes experienced and biographic memories that are defining features of episodic memory, and the pre-frontal cortex suppresses context-inappropriate memories thus allowing the retrieval of context-appropriate memories. (7) After brain trauma, cognitive impairment might be persistent (8) and no available treatments have been shown to be effective to improve those sequels.

Non-invasive brain stimulation (NIBS) techniques, including transcranial direct current stimulation (tDCS), are neuromodulatory interventions that have been shown to improve neuroplasticity and cognitive outcomes in neurological conditions, including TBI. (9,10) tDCS can transiently alter neuronal activity facilitating or inhibiting neuronal circuitries depending on the polarity of the stimulation. (11) tDCS induces neuroplasticity by applying a low-intensity electrical current (0.5 - 2 mA) through electrodes placed on the scalp. The electrodes have two polarities (anode and cathode) and change the resting state of the membrane cells of the surrounding region. (12,13) Previous studies have shown that repetitive tDCS sessions improved disorder of consciousness (11,14,15) and cognition in patients with TBI, (8,16) whereas some studies have shown inconsistent results. (17–19)

For peer review only

Since tDCS works on the membrane level, changing the resting state but not evoking action potential, the use of tDCS with concurrent cognitive training seems to be a good option to potentiate the stimulation and modulate the brain networks accordingly to the target training. (20–23) Two prior studies investigated the effects of the use of tDCS and cognitive training (non-concurrent) on memory and attention performance in TBI patients, but only one found a significant improvement in the cognitive outcome measures. (20,22)

Biomarkers that evaluate brain changes after the tDCS intervention are still scarce, however the electroencephalogram (EEG) has been suggested to be a useful tool for this purpose. (24–29) The EEG measures the rhythm of electrical activity in the brain according to its frequency: Delta (1 - 4Hz), Theta (4 - 8Hz), Alpha (8 - 12Hz),Beta (12 - 30Hz) and Gamma (30 - 40Hz) (27,30,31) and is widely used as a safety outcome in patients who undergo tDCS sessions. (32–35) Some studies associate EEG measures (amplitude, power, phase and coherence) to the functionality of patients, (36) including the diagnosis and prognosis of patients with TBI. (37,38) A study using EEG power spectrum (39) suggests that, after 10 tDCS sessions, changes in the rhythm of brain activity occur, with reduction of delta and increase of alpha near the active electrodes in patients with chronic TBI. This study also found a significant correlation between decreases in delta and improved performance on neuropsychological tests for the active tDCS group to far greater extent than for the sham group. (24) Other studies have measured cortical activity after a single session of tDCS and have shown inconsistent results. (24–29) Thus, cortical changes after consecutive sessions of tDCS combined with cognitive training in people with TBI are still inconclusive.

Due to the lack of consensus and scarcity of evidence about the effects of cognitive training in addition to tDCS sessions in patients with TBI, the goals of this study are (a)

For peer review only

to investigate the effect of 10 sessions of tDCS and concurrent cognitive training in patients with TBI compared to sham tDCS; (b) to analyze differences on episodic memory scores between active anodal tDCS over the left dorsolateral prefrontal cortex (lDLPF) and bilateral temporal cortex (BTC); (c) to analyze changes on cortical activities (measured by the EEG) between the groups. We hypothesize that (a) participants that received active stimulation will have greater scores on episodic memory test compared to the sham group; (b) there might be significant score differences on episodic memory test between patients who were stimulated over the BTC and those stimulated over the lDPFC; (c) delta reduction and an increase in alpha waves close to the sponge placement in the active group compared to the sham group.

## METHODS AND ANALYSIS

### Design

This is a randomized, parallel-group, placebo-controlled and double-blind study that is being conducted at Hospital das Clínicas, University of São Paulo, (HC-FMUSP), São Paulo, Brazil. Participants who meet eligibility criteria are randomly allocated to (1) Group 1 - bilateral temporal cortex (BTC); (2) Group 2 - left dorsolateral prefrontal cortex (lDLPFC); (3) Group 3 – sham (BTC/lDLPFC).

Twenty-minute-tDCS for 10 days (2 weeks, except for Saturdays and Sundays) is delivered simultaneously to a computer-assisted cognitive training. Patients will be assessed at baseline (T0), at the end of the last stimulation session (T1) and three months after the last tDCS session (T2). (Figure 1).

### Ethics committee and regulatory approval

The trial is conducted in accordance with the ethical principles outlined in the Declaration of Helsinki, 1996. This research was approved by the Hospital das Clínicas,

For peer review only

University of São Paulo Ethical Institutional Review Border number CAAE: 87954518.0.0000.0068. Clinical Trial number NCT04540783. Any severe side effect during the trial will be reported to the safety monitoring board IRB for appropriate management.

**Randomization and blinding**

The investigator ALZ was responsible for the computer-generated random assignment list, arranging patients in blocks of 3 or 6. The proportion of the randomization for each group is 1:1:1. This randomized list ensures double blinding so that both research assistants and patients are blind to the type of stimulation. Before each stimulation session, the researcher responsible for the stimulation receives a code that allows the tDCS device to deliver 20 minutes of active or sham stimulation. This blinding and methodological procedure is similar to the rational of previous studies. (20,22,24)

The randomization list and the Neuroconn (tDCS device) code is kept inside a locked drawer with restricted access at the research coordination office at Hospital das Clínicas (HC-FMUSP).

**Recruitment and study population**

Thirty-six patients with TBI are being recruited from hospitals in São Paulo. All participants provide written informed consent and receive an exclusive identification number during the screening period, to ensure blinding. Study recruitment started in June 2019 and the estimated completion date for the primary outcome is June 2022. We expect that 85% of the patients will be inpatients from HC-FMUSP referred by neurologists and 15% from extra-mural recruitment (from social media and folders). This trial follows the CONSORT (Consolidated Standards of Reporting Trials) guidelines.

For peer review only

**Inclusion criteria**

• Outpatients with radiological diagnosis of TBI at least 6 months prior to enrollment in the study.

• Glasgow Coma Scale (GCS) score ≤ 12 at admission in the emergency room.

• Memory complaints, self-reported or reported by the family/ caregiver.

• Age between 18 and 55 years.

• Able to follow directions.

**Exclusion criteria**

• History of epilepsy post-TBI.

• Clinical EEG abnormalities (epileptiform activity, disorganized background , in other words, a general change in the way a normal brain wave looks – frequency, height and shape).

• Uncorrected visual impairment.

• Contraindications to tDCS, such as medical devices implanted in the brain or metallic foreign body in the head.

• Current severe/major depression (score over 36 points on the Beck Depression Inventory – 2nd edition).

• Current severe anxiety (score over 26 points on the Back Anxiety Inventory).

• Limiting motor deficit.

• Estimated IQ under 70.

• Time after trauma > 18 months

**Patient and public involvement**

Patients are not involved in the design, or conduct, or reporting, or dissemination plans of our research.

For peer review only

**Instruments**

Patients are expected to come to the research hospital for 11 visits as described in Table 1.

Table 1: Detail of the study visits.

| | Tasks | Visit 1 Baseline | Visit 2-9 | Visit 10 | Visit 11 3 months follow-up |
|---|---|---|---|---|---|
| Consent Form | X | | | | |
| Screening | | | | | |
| Medical history | X | | | | |
| qEEG | X | | | X | X |
| BDI-II | X | | | X | X |
| BAI | X | | | X | X |
| Estimated IQ | X | | | | |
| Episodic Memory | | | | | |
| RAVLT | | X | | X | X |
| Intervention | | | | | |
| tDCS | | X | X | X | |
| Cognitive Training | | X | X | X | |
| AEQ | | X | X | X | |

Legend: AEQ – Adverse Events Questionnaire; BAI - Beck Anxiety Inventory; BDI-II - Beck Depression Inventory; qEEG – Quantitative Electroencephalogram; IQ – Intelligence quotient, tDCS – Transcranial Direct Current Stimulation, RAVLT - Rey Auditory Verbal Learning Test.

**Screening assessment:**

Depressive symptoms - BDI-II - Beck Depression Inventory (40)

Anxiety symptoms - BAI - Beck Anxiety Inventory (41)

Estimated IQ - WAIS - Wechsler Adult Intelligence Scale (Matrix Reasoning and Vocabulary) (42,43).

**Primary outcome (Episodic Memory)**

Rey Auditory Verbal Learning Test (RAVLT) - A list of 15 words is presented, and subjects are asked to say all words they can remember. The process is repeated 5 times. Twenty minutes after the 5th trial, an interference list of 15 words (List B) is presented, followed by a free-recall test of that list. Immediately after this, delayed recall of the first list is tested (Trial 6) without further presentation of those words. After a 20-

For peer review only

min delay period, subjects are again required to recall words from List A (Trial 7). Finally, recognition can be tested with the use of a matrix array, in which the individual must identify List A words from a list of 50 words containing all items from Lists A and B and 20 words that are phonemically or semantically similar to those in Lists A and B. (44) The 7th trial of the list A will be used as primary outcome.

**Secondary outcome**

EEG assessment. The exam is performed on the Nihon Kohden® EEG 1200 version 01.71 digital equipment, with simultaneous video recording with a Sony® Ipela camera. For qualitative EEG data collection, we use the international 10-20 electrode placement system, 19 channels (being one to electrocardiogram), with sampling rate of 200Hz, a time of 0.3, high filter from 35 to 70 Hz and sensitivity of 7 μV. For the quantitative analysis, the data are converted using Neuromap from the Neuroworkbench software. The exam lasts 30 minutes (15 minutes with your eyes open and 15 minutes with your eyes closed - relaxed wakefulness). The analyses are performed by a certified neurophysiologist (STS).

**Safety screening**

Adverse Events Questionnaire (AEQ): Questionnaire that must be answered after each stimulation session to assess adverse effects such as tingling sensations, itching, mild transient redness of the skin and discomfort on the region of stimulation, moderate fatigue, difficulty concentrating, headache, and nauseas. (45)

**Intervention**

*Transcranial direct current stimulation (tDCS)*

Both anodal and sham tDCS will be delivered by the same battery-driven (NeuroConn: DC Stimulator Plus), for 20 minutes. The research assistant will set up the

For peer review only

device according to the assignment list in order of participant's registration number. Saline-soaked surface 35cm² (5cm X 7cm) sponge electrodes connected to the stimulator will be placed upon the patient's scalp and secured with adjustable rubber straps.

The sponge placement follows the 10-20 EEG system. Group 1 - bilateral temporal cortex (BTC) - two anode electrodes are placed over T3 and T4 respectively, and the cathode electrode over the supraorbital region (FP2). Group 2 – left dorsolateral prefrontal cortex (lDLPFC), the anode electrode is placed over F3 and the cathode over FP2. Group 3 – sham group – half of the participants are following the montage of group 1 (BTC) and the other half from group 2 (lDLPFC). T3, T4 and F3 regions have been chosen for this protocol because other studies have investigated the effects of tDCS on memory by placing the electrodes over those regions. (46–55) For the sham stimulation, patients receive the active current with ramping up and down for 30 seconds to simulate the real stimulation over the BTC or lDLPFC, as referred by other studies. (56,57) Patients are monitored daily for side effects, according to international safety guidelines, and with the Adverse Events Questionnaire (AEQ). (45)

*Cognitive Training*

The Rehacom is a cognitive software for patients with different etiologies approved by the Brazilian Health Regulatory Agency (ANVISA). This software has several cognitive modules. For the purpose of the present study, we are using the attentional visual and verbal memory training tasks with increasing levels of difficulty according to the patient's performance. During the training, the feedback option is active, so the patient can be oriented and improve his/her performance over the trials. The initial level is adjusted to level 1 for all patients who have incomplete high school, for those who complete high school the starting level is 4, and for those with complete college, the starting level is 5. (58–60)

For peer review only

The cognitive training follows two possible random sequence order – memory/ attention or attention/memory modules, always alternating daily up to the end of the last stimulation session. Each training has a 20-minute duration, always combined with the tDCS.

**Sample size calculation**

The sample calculation was performed using the software GPower 3.1, using the statistical 2-wayANOVA (3 groups and 3 timepoints), a given α 5%, power 80% and, interaction effect of 0.25 considering the primary outcome, based on our pilot data. G power analysis provided a sample size of 36 participants based on the F calculation (12 patients per group).

**Statistical analysis**

Descriptive statistics are used to report demographic data. Kolmogorov-Smirnov test was used to test data normality. To analyze the primary outcome, changes on episodic memory (RAVLT scores), we will use ANOVA for normal data distribution or non-parametric tests. We assume that each participant has a random effect on the model. For the secondary outcome (EEG spectral power), we plan to use the mixed effect model (reml), considering group and time as fixed factors and each participant as a random effect. Estimated alpha value of 5%. An intention-to-treat framework will be applied.

**Ethics and dissemination**

TDCS is a safe intervention not only because the electric current applied is very low (0.5 - 2mA over a 25-35 cm² area), but also because the electrodes embedded in saline solution minimize tissue resistance, avoid overheating. Tingling sensations, itching, mild transient redness of the skin and discomfort on the region of stimulation, moderate fatigue, difficulty concentrating, headache and nauseas are possible adverse effects, but these

For peer review only

effects do not usually last long and are often seen at the same frequency in experimental and placebo groups. (10,61,62) The safety side-effect questionnaire (AEQ (45)) is collected after each stimulation session. Complaints regarding the stimulation or high AEQ scores are reported to the safety board and the medical coverage may be called for necessary care. Written informed consent for participation in the study will be obtained from all participants. Participant information is stored securely in locked file cabinets and participant digital information is password protected.

## DISCUSSION

In order to contribute to the development of evidence-based rehabilitation treatments to TBI survivors with memory impairments, the present study aims to investigate whether the use of tDCS targeting the BTC or the lDLFFC with concurrent computer-based cognitive training improves memory performance in patients with moderate and severe closed TBI patients.

Since TBI causes health loss and disability for individuals and their families (63) and memory impairment is one of the most frequent cognitive complaints, (64,65) an effective rehabilitation tool will be helpful to improve this burden in this population.

There is evidence that tDCS may improve cognitive impairments, such as memory impairments, following TBI and other etiologies. (49,50) Prior research has shown the efficacy of anodal tDCS in improving memory performance during tasks such as face-name associative recall tasks, intentional memorization of words, figure-naming tests, word recall and picture-pseudoword associative learning tasks. (51–55) A recent systematic review found 14 experimental studies on adult TBI patients who received tDCS for the assessment of clinical or surrogate outcomes (66) and, to our knowledge, only two studies used tDCS concomitant to cognitive training (non-concurrent) in TBI patients. (20–22)

For peer review only

Despite some disadvantages, namely poor spatial/temporal resolution and stimulation of large part of the brain, there are many advantages to tDCS, such as low risk of adverse effects and low cost. (67) It has been proven that tDCS does not induce depolarization, meaning it does not induce the firing of neurons when they are not near threshold. Therefore, it is less likely that neurons not engaged in the task at hand will discharge, hence the importance of applying tDCS during a specific task in order to target a particular circuitry. (23) It has also been suggested that more systematic investigations are needed, due to the heterogeneity of findings in tDCS research and the different parameters used in the stimulation. (52,68)

EEG will be used to guarantee safety (32–35) and to measure cortical activity post intervention. Spikes and abnormal waves shown on the EEG will provide clinical guidance on whether to include the participant in the present study. We expect to reduce delta activities and increase alpha frequencies close to the active electrodes and find a better performance correlation in neuropsychological tests in the active group, as demonstrated previously. (24)

One limitation of this study is that, due to sample size restrictions, sex and TBI severity will not be considered as covariates. Severe TBI and moderate TBI are considered as a single entity for investigation purposes in many studies, in part because of the permanent physical, cognitive and behavioral impairments that are observed in such patients in comparison to mild TBI patients. As for sex differences, a recent study aimed at characterizing the demographic, social and economic profile of TBI patients in Brazil showed that men were hospitalized almost 3.5 times more frequently for TBI than women and that the incidence of TBI in the male population was 102/100,000/year. (69–71)

For peer review only

This is a study to test the effectiveness of combined tDCS and cognitive training to improve episodic memory in patients with TBI. The results generated may potentially be useful for other neurological disorders that cause cognitive impairments. Our open-label pilot study (n=4 participants) has proven the feasibility of the method and a moderate effect size of the RAVLT scores between the baseline to the last tDCS session. Results will be presented at conferences and submitted for publication in peer-reviewed journals.

**Trial Status**

This trial was registered on the website clinicaltrials.gov with the registration number NCT04540783. The open-label pilot study was performed with 4 participants in 2018 and validated the study protocol. Recruitment started in February 2019. At the time of submission of this paper, we had completed 15 participants. The programmed completion date for the primary outcome is June 2022.

This study will provide important data regarding the use of the combined tools to improve the memory of persons that suffer from the sequela of a TBI. Larger clinical trial studies are required to further interrogate the clinical efficacy of this technique to improve the mood and the quality of life of this target population.

**Acknowledgements**

The authors thank the Institute of Psychiatry, Hospital das Clínicas (IPq HCFMUSP), the secretary Sandra Aparecida de Lima Falcon and all participants and their families for their support and assistance.

**Conflicts of Interests**

Authors report no conflict of interests.

For peer review only

**Data**

Technical appendix, statistical code, and dataset will be available from the following link: https://drive.google.com/file/d/1WLV4yowqcyU5DuHW9PBI3FQtNrS2WKWe/view?usp=sharing

**Author Contributions**

Research project: Conception – ALZ, WSP.

Organization – ALZ, WSP, ARB, LV, VMG.

Execution – DJF, VMP, DC, STS.

Statistical Analysis: Design – ALZ, WSP, ARB

Manuscript Preparation: First draft – DJF. Final draft – DJF, VMP, DC, ALZ

Review and Critique: ALZ, ARB, LV, VMG, DC, DJF, VMP.

**Funding**

Vanessa M. Paglioni reports grant from Coordination for the Improvement of Higher Education Personnel (CAPES) – Brazil.

Dr. Ana Luiza Zaninotto is supported by the National Institute of Health 3R01DC017291 – 02.

Dr. Wellingson Silva Paiva is supported by the NIHR Global Health Research Group on Neurotrauma, which was commissioned by the National Institute for Health Research (NIHR) using UK aid from the UK Government (project 16/137/105).

The views expressed in this publication are those of the author(s) and not necessarily those of the NIH, NIDCD, NIHR or the Department of Health and Social Care.

**ORCID IDs**

Dagliê Jorge de Freitas https://orcid.org/0000-0002-2473-6210

Daniel de Carvalho https://orcid.org/0000-0003-4960-0800

Vanessa M. Paglioni https://orcid.org/0000-0002-8008-2231

For peer review only

Andre R. Brunoni https://orcid.org/0000-0002-6310-3571

Leandro Valiengo https://orcid.org/0000-0002-7628-9993

Sigride Thome-Souza https://orcid.org/0000-0002-3017-6723

Vinicius Monteiro de Paula Guirado https://orcid.org/0000-0002-7782-3609

Ana Luiza Zaninotto https://orcid.org/0000-0003-0639-0612

Wellingson S. Paiva https://orcid.org/0000-0002-5165-7040

**Legend for Figure 1:** Figure 1 CONSORT Flow Diagram

For peer review only

## REFERENCES


1. Menon DK, Schwab K, Wright DW, Maas AI. Position statement: Definition of traumatic brain injury. Vol. 91, Archives of Physical Medicine and Rehabilitation. W.B. Saunders; 2010. p. 1637–40.
2. Gentleman SM, Roberts GW, Gennarelli TA, Maxwell WL, Adams JH, Kerr S, et al. Axonal injury: a universal consequence of fatal closed head injury? Acta Neuropathol. 1995;89(6):537–43.
3. Miotto EC, Cinalli FZ, Serrao VT, Benute GG, Lucia MCS, Scaff M. Cognitive deficits in patients with mild to moderate traumatic brain injury. Arq Neuropsiquiatr [Internet]. 2010 [cited 2020 Jun 25];68(6):862–8. Available from: http://www.scielo.br/scielo.php?script=sci_arttext&pid=S0004-282X2010000600006&lng=en&nrm=iso&tlng=en
4. Wright MJ, Schmitter-Edgecombe M. The impact of verbal memory encoding and consolidation deficits during recovery from moderate-to-severe traumatic brain injury. J Head Trauma Rehabil. 2011;26(3):182–91.
5. Vakil E, Greenstein Y, Weiss I, Shtein S. The Effects of Moderate-to-Severe Traumatic Brain Injury on Episodic Memory: a Meta-Analysis. Neuropsychol Rev. 2019;29(3):270–87.
6. Moscovitch M, Cabeza R, Winocur G, Nadel L. Episodic Memory and Beyond: The Hippocampus and Neocortex in Transformation. Annu Rev Psychol. 2016;67(1):105–34.
7. Eichenbaum H. Prefrontal-hippocampal interactions in episodic memory. Nat Rev Neurosci [Internet]. 2017;18(9):547–58. Available from: http://dx.doi.org/10.1038/nrn.2017.74
8. Ponsford JL, Downing MG, Olver J, Ponsford M, Acher R, Carty M, et al. Longitudinal follow-up of patients with traumatic brain injury: Outcome at two, five, and ten years post-injury. J Neurotrauma. 2014;31(1):64–77.
9. Michael A. Nitsche, David Liebetanz NL, Andrea Antal, Frithjof Tergau WP. Safety criteria for transcranial direct current stimulation (tDCS) in humans. Clin Neurophysiol. 2003;114:2220–2.
10. Nitsche MA, Cohen LG, Wassermann EM, Priori A, Lang N, Antal A, et al. Transcranial direct current stimulation: State of the art 2008. Brain Stimul. 2008;1(3):206–23.
11. Levin HS. Memory deficit after closed-head injury. J Clin Exp Neuropsychol. 1990;12(1):129–53.
12. Li S, Zaninotto AL, Neville IS, Paiva WS, Nunn D, Fregni F. Clinical utility of brain stimulation modalities following traumatic brain injury: Current evidence. Neuropsychiatr Dis Treat. 2015;11:1573–86.
13. Amatachaya A, Auvichayapat N, Patjanasoontorn N, Suphakunpinyo C, Ngernyam N, Aree-uea B, et al. Effect of Anodal Transcranial Direct Current Stimulation on Autism: A Randomized Double-Blind Crossover Trial. Behav Neurol. 2014;2014:1–7.
14. Palacios EM, Sala-Llonch R, Junque C, Fernandez-Espejo D, Roig T, Tormos JM, et al. Long-term declarative memory deficits in diffuse TBI: Correlations with cortical thickness, white matter integrity and hippocampal volume. Cortex [Internet]. 2013;49(3):646–57. Available from: http://dx.doi.org/10.1016/j.cortex.2012.02.011
15. Sozda CN, Muir JJ, Springer US, Partovi D, Cole MA. Differential learning and memory performance in OEF/OIF veterans for verbal and visual material.


For peer review only

Neuropsychology. 2014;28(3):347–52.
16. Zaninotto AL, Vicentini JE, Solla DJF, Silva TT, De Paula Guirado VM, Feltrin F, et al. Visuospatial memory improvement in patients with diffuse axonal injury (DAI): A 1-year follow-up study. Acta Neuropsychiatr. 2017;29(1):35–42.
17. Estraneo A, Pascarella A, Moretta P, Masotta O, Fiorenza S, Chirico G, et al. Repeated transcranial direct current stimulation in prolonged disorders of consciousness: A double-blind cross-over study. J Neurol Sci [Internet]. 2017;375:464–70. Available from: http://dx.doi.org/10.1016/j.jns.2017.02.036
18. Wilke S, List J, Mekle R, Lindenberg R, Bukowski M, Ott S, et al. No effect of anodal tDCS on GABA levels in patients with recurrent mild traumatic brain injury. J Neurotrauma. 2016;1–50.
19. Kang EK, Kim DY, Paik NJ. Transcranial direct curent stimulation of the left prefrontal cortex improves attention in patients with traumatic brain injury: A pilot study. J Rehabil Med. 2012;44(4):346–50.
20. Leśniak M, Polanowska K, Seniów J, Członkowska A. Effects of repeated anodal tDCS coupled with cognitive training for patients with severe traumatic brain injury: A pilot randomized controlled trial. J Head Trauma Rehabil. 2014;29(3).
21. Galetto V, Sacco K. Neuroplastic Changes Induced by Cognitive Rehabilitation in Traumatic Brain Injury: A Review. Neurorehabil Neural Repair. 2017;31(9):800–13.
22. Sacco K, Galetto V, Dimitri D, Geda E, Perotti F, Zettin M, et al. Concomitant use of transcranial direct current stimulation and computer-assisted training for the rehabilitation of attention in traumatic brain injured patients: Behavioral and neuroimaging results. Front Behav Neurosci. 2016;10(MAR):1–11.
23. Miniussi C, Harris JA, Ruzzoli M. Modelling non-invasive brain stimulation in cognitive neuroscience. Neurosci Biobehav Rev [Internet]. 2013;37(8):1702–12. Available from: http://dx.doi.org/10.1016/j.neubiorev.2013.06.014
24. Ulam F, Shelton C, Richards L, Davis L, Hunter B, Fregni F, et al. Cumulative effects of transcranial direct current stimulation on EEG oscillations and attention/working memory during subacute neurorehabilitation of traumatic brain injury. Clin Neurophysiol [Internet]. 2015;126(3):486–96. Available from: http://dx.doi.org/10.1016/j.clinph.2014.05.015
25. Keeser D, Padberg F, Reisinger E, Pogarell O, Kirsch V, Palm U, et al. Prefrontal direct current stimulation modulates resting EEG and event-related potentials in healthy subjects: A standardized low resolution tomography (sLORETA) study. Neuroimage [Internet]. 2010;55(2):644–57. Available from: http://dx.doi.org/10.1016/j.neuroimage.2010.12.004
26. Balconi M, Vitaloni S. The tDCS effect on alpha brain oscillation for correct vs. incorrect object use. The contribution of the left DLPFC. Neurosci Lett [Internet]. 2012;517(1):25–9. Available from: http://dx.doi.org/10.1016/j.neulet.2012.04.010
27. Maeoka H, Matsuo A, Hiyamizu M, Morioka S, Ando H. Influence of transcranial direct current stimulation of the dorsolateral prefrontal cortex on pain related emotions: A study using electroencephalographic power spectrum analysis. Neurosci Lett [Internet]. 2012;512(1):12–6. Available from: http://dx.doi.org/10.1016/j.neulet.2012.01.037
28. Jacobson L, Ezra A, Berger U, Lavidor M. Modulating oscillatory brain activity correlates of behavioral inhibition using transcranial direct current stimulation. Clin Neurophysiol [Internet]. 2012;123(5):979–84. Available from: http://dx.doi.org/10.1016/j.clinph.2011.09.016

For peer review only

29. Miller J, Berger B, Sauseng P. Anodal transcranial direct current stimulation (tDCS) increases frontal-midline theta activity in the human EEG: A preliminary investigation of non-invasive stimulation. Neurosci Lett [Internet]. 2015;588:114–9. Available from: http://dx.doi.org/10.1016/j.neulet.2015.01.014
30. St. Louis E, Frey L, Britton J, Hopp J, Korb P, Koubeissi M, et al. Electroencephalography (EEG): An Introductory Text and Atlas of Normal and Abnormal Findings in Adults, Children, and Infants. Electroencephalography (EEG): An Introductory Text and Atlas of Normal and Abnormal Findings in Adults, Children, and Infants. 2016. 1–96 p.
31. Hughes J. EEG in clinical practice. 2nd ed. Washington Street Newton: Butterworth-Heinemann; 1994. 242 p.
32. Liu A, Bryant A, Jefferson A, Friedman D, Minhas P, Barnard S, et al. Exploring the efficacy of a 5-day course of transcranial direct current stimulation (TDCS) on depression and memory function in patients with well-controlled temporal lobe epilepsy. Epilepsy Behav. 2016;55:11–20.
33. Antal A, Boros K, Poreisz C, Chaieb L, Terney D, Paulus W. Comparatively weak after-effects of transcranial alternating current stimulation (tACS) on cortical excitability in humans. Brain Stimul. 2008;1(2):97–105.
34. Zaehle T, Sandmann P, Thorne JD, Jäncke L, Herrmann CS. Transcranial direct current stimulation of the prefrontal cortex modulates working memory performance: Combined behavioural and electrophysiological evidence. BMC Neurosci. 2011;12:9–14.
35. Tadini L, El-Nazer R, Brunoni AR, Williams J, Carvas M, Boggio P, et al. Cognitive, mood, and electroencephalographic effects of noninvasive cortical stimulation with weak electrical currents. J ECT. 2011;27(2):134–40.
36. Hannawi Y, Stevens RD. Mapping the connectome following traumatic brain injury. Curr Neurol Neurosci Rep. 2016;16(5).
37. Marceglia S, Mrakic-Sposta S, Rosa M, Ferrucci R, Mameli F, Vergari M, et al. Transcranial direct current stimulation modulates cortical neuronal activity in Alzheimer's disease. Front Neurosci. 2016;10(MAR):1–11.
38. Roche RAP, Dockree PM, Garavan H, Foxe JJ, Robertson IH, O'Mara SM. EEG alpha power changes reflect response inhibition deficits after traumatic brain injury (TBI) in humans. Neurosci Lett. 2004;362(1):1–5.
39. Dressler O, Schneider G, Stockmanns G, Kochs EF. Awareness and the EEG power spectrum: Analysis of frequencies. Br J Anaesth. 2004;93(6):806–9.
40. Gorenstein C, Wang YP, Argimon I de L, Werlang BSG. Manual do Inventário de depressão de Beck-BDI-II. 1st ed. São Paulo: Editora Casa do Psicólogo; 2011. 172 p.
41. Cunha JA. Manual da versão em português das Escalas Beck. 1st ed. Casa do Psicólogo; 2001. 171 p.
42. Denney DA, Ringe WK, Lacritz LH. Dyadic short forms of the Wechsler Adult Intelligence Scale-IV. Arch Clin Neuropsychol. 2015;30(5):404–12.
43. Wechsler D. WAIS-III: Escala de inteligência Wechsler para adultos: manual técnico. 1st ed. São Paulo: Casa do Psicólogo; 2004. 276 p.
44. De Paula J, Fernandes Malloy-Diniz L. Teste de Aprendizagem Auditivo-Verbal de Rey (RAVLT). São Paulo: Vetor Editora Psico-Pedagógica Ltda.; 2018.
45. Brunoni AR, Amadera J, Berbel B, Volz MS, Rizzerio BG, Fregni F. A systematic review on reporting and assessment of adverse effects associated with transcranial direct current stimulation. Int J Neuropsychopharmacol. 2011;14(8):1133–45.

46. Ross LA, McCoy D, Coslett HB, Olson IR, Wolk DA. Improved proper name recall in aging after electrical stimulation of the anterior temporal lobes. Front Aging Neurosci. 2011;3(OCT):1–8.
47. Jeong Yun G, Ho Chun M, Ryun Kim B. The Effects of Transcranial Direct-Current Stimulation on Cognition in Stroke Patients. 2015; Available from: http://j-stroke.org
48. Boggio PS, Khoury LP, Martins DCS, Martins OEMS, De Macedo EC, Fregni F. Temporal cortex direct current stimulation enhances performance on a Visual recognition memory task in Alzheimer disease. J Neurol Neurosurg Psychiatry. 2009;80(4):444–7.
49. Sandrini M, Brambilla M, Manenti R, Rosini S, Cohen LG, Cotelli M. Noninvasive stimulation of prefrontal cortex strengthens existing episodic memories and reduces forgetting in the elderly. Front Aging Neurosci. 2014;6(OCT):1–9.
50. Park SH, Koh EJ, Choi HY, Ko MH. A double-blind, sham-controlled, pilot study to assess the effects of the concomitant use of transcranial direct current stimulation with the computer assisted cognitive rehabilitation to the prefrontal cortex on cognitive functions in patients with strok. J Korean Neurosurg Soc. 2013;54(6):484–8.
51. Leshikar ED, Leach RC, McCurdy MP, Trumbo MC, Sklenar AM, Frankenstein AN, et al. Transcranial direct current stimulation of dorsolateral prefrontal cortex during encoding improves recall but not recognition memory. Neuropsychologia [Internet]. 2017;106:390–7. Available from: https://doi.org/10.1016/j.neuropsychologia.2017.10.022
52. Medvedeva A, Materassi M, Neacsu V, Beresford-Webb J, Hussin A, Khan N, et al. Effects of Anodal Transcranial Direct Current Stimulation Over the Ventrolateral Prefrontal Cortex on Episodic Memory Formation and Retrieval. Cereb Cortex. 2019;29(2):657–65.
53. Fileccia E, Di Stasi V, Poda R, Rizzo G, Stanzani-Maserati M, Oppi F, et al. Effects on cognition of 20-day anodal transcranial direct current stimulation over the left dorsolateral prefrontal cortex in patients affected by mild cognitive impairment: a case-control study. Neurol Sci. 2019;40(9):1865–72.
54. Sandrini M, Manenti R, Gobbi E, Rusich D, Bartl G, Cotelli M. Transcranial direct current stimulation applied after encoding facilitates episodic memory consolidation in older adults. Neurobiol Learn Mem [Internet]. 2019;163(January):107037. Available from: https://doi.org/10.1016/j.nlm.2019.107037
55. Antonenko D, Thams F, Uhrich J, Dix A, Thurm F, Li SC, et al. Effects of a multi-session cognitive training combined with brain stimulation (TRAINSTIM-COG) on age-associated cognitive decline – Study protocol for a randomized controlled phase IIb (MONOCENTER) trial. Front Aging Neurosci. 2019;10(JUL):1–12.
56. Bikson M, Grossman P, Thomas C, Zannou AL, Jiang J, Adnan T, et al. Safety of Transcranial Direct Current Stimulation: Evidence Based Update 2016. Brain Stimul [Internet]. 2016;9(5):641–61. Available from: http://dx.doi.org/10.1016/j.brs.2016.06.004
57. Antal A, Alekseichuk I, Bikson M, Brockmöller J, Brunoni AR, Chen R, et al. Low intensity transcranial electric stimulation: Safety, ethical, legal regulatory and application guidelines. Clin Neurophysiol [Internet]. 2017;128(9):1774–809. Available from: http://dx.doi.org/10.1016/j.clinph.2017.06.001

For peer review only

58. Fernandez E, Bergado Rosado JA, Perez DR, Santana SS, Aguilar MT, Bringas ML. Effectiveness of a computer-based training program of attention and memory in patients with acquired brain damage. Behav Sci (Basel). 2018;8(1).
59. Yoo C, Yong MH, Chung J, Yang Y. Effect of computerized cognitive rehabilitation program on cognitive function and activities of living in stroke patients. J Phys Ther Sci. 2015;27(8):2487–9.
60. Veisi-Pirkoohi S, Hassani-Abharian P, Kazemi R, Vaseghi S, Zarrindast MR, Nasehi M. Efficacy of RehaCom cognitive rehabilitation software in activities of daily living, attention and response control in chronic stroke patients. J Clin Neurosci [Internet]. 2020;71:101–7. Available from: https://doi.org/10.1016/j.jocn.2019.08.114
61. Brunoni A, Nitsche M, Bolognini N, Bikson M, Wagner T, Merabet L, et al. Clinical research with tDCS: Challenges and future directions. Brain Stimul. 2012;5(3):175–95.
62. de Amorim RLO, Brunoni AR, de Oliveira MAF, Zaninotto ALC, Nagumo MM, Guirado VM de P, et al. Transcranial direct current stimulation for post-concussion syndrome: Study protocol for a randomized crossover trial. Front Neurol. 2017 May 2;8(MAY):164.
63. James SL, Theadom A, Ellenbogen RG, Bannick MS, Montjoy-Venning W, Lucchesi LR, et al. Global, regional, and national burden of traumatic brain injury and spinal cord injury, 1990–2016: a systematic analysis for the Global Burden of Disease Study 2016. Lancet Neurol. 2019 Jan 1;18(1):56–87.
64. Jourdan C, Bayen E, Pradat-Diehl P, Ghout I, Darnoux E, Azerad S, et al. A comprehensive picture of 4-year outcome of severe brain injuries. Results from the PariS-TBI study. Ann Phys Rehabil Med [Internet]. 2016;59(2):100–6. Available from: http://dx.doi.org/10.1016/j.rehab.2015.10.009
65. Zec RF, Zellers D, Belman J, Miller J, Matthews J, Ferneau-Belman D, et al. Long-term consequences of severe closed head injury on episodic memory. J Clin Exp Neuropsychol. 2001;23(5):671–91.
66. Zaninotto AL, El-Hagrassy MM, Green JR, Babo M, Paglioni VM, Guerra Benute G, et al. Transcranial direct current stimulation (tDCS) effects on traumatic brain injury (TBI) recovery A systematic review. Dement Neuropsychol [Internet]. 2019 [cited 2019 Jul 2];13(2):172–9. Available from: http://dx.doi.org/10.1590/1980-57642018dn13-020005
67. Miniussi C, Ruzzoli M. Transcranial stimulation and cognition. In: Lozano AM, Hallett M, editors. Handbook of Clinical Neurology [Internet]. 1st ed. Elsevier B.V.; 2013. p. 739–50. Available from: http://dx.doi.org/10.1016/B978-0-444-53497-2.00056-5
68. Galli G, Vadillo MA, Sirota M, Feurra M, Medvedeva A. A systematic review and meta-analysis of the effects of transcranial direct current stimulation (tDCS) on episodic memory. Brain Stimul [Internet]. 2019;12(2):231–41. Available from: https://doi.org/10.1016/j.brs.2018.11.008
69. Strickland TL, Depalma RG, Golanov E, Timmer ML, Jacobs B, Schonherr MC, et al. The Spectrum of Long-Term Behavioral Disturbances and Provided Care After Traumatic Brain Injury. Front Neurol | www.frontiersin.org [Internet]. 2020;1:246. Available from: www.frontiersin.org
70. Azouvi P, Arnould A, Dromer E, Vallat-Azouvi C. Neuropsychology of traumatic brain injury: An expert overview. Vol. 173, Revue Neurologique. Elsevier Masson SAS; 2017. p. 461–72.
71. De Almeida CER, De Sousa Filho JL, Dourado JC, Gontijo PAM, Dellaretti MA,


For peer review only

Costa BS. Traumatic Brain Injury Epidemiology in Brazil. World Neurosurg [Internet]. 2016;87:540–7. Available from: http://dx.doi.org/10.1016/j.wneu.2015.10.020

For peer review only

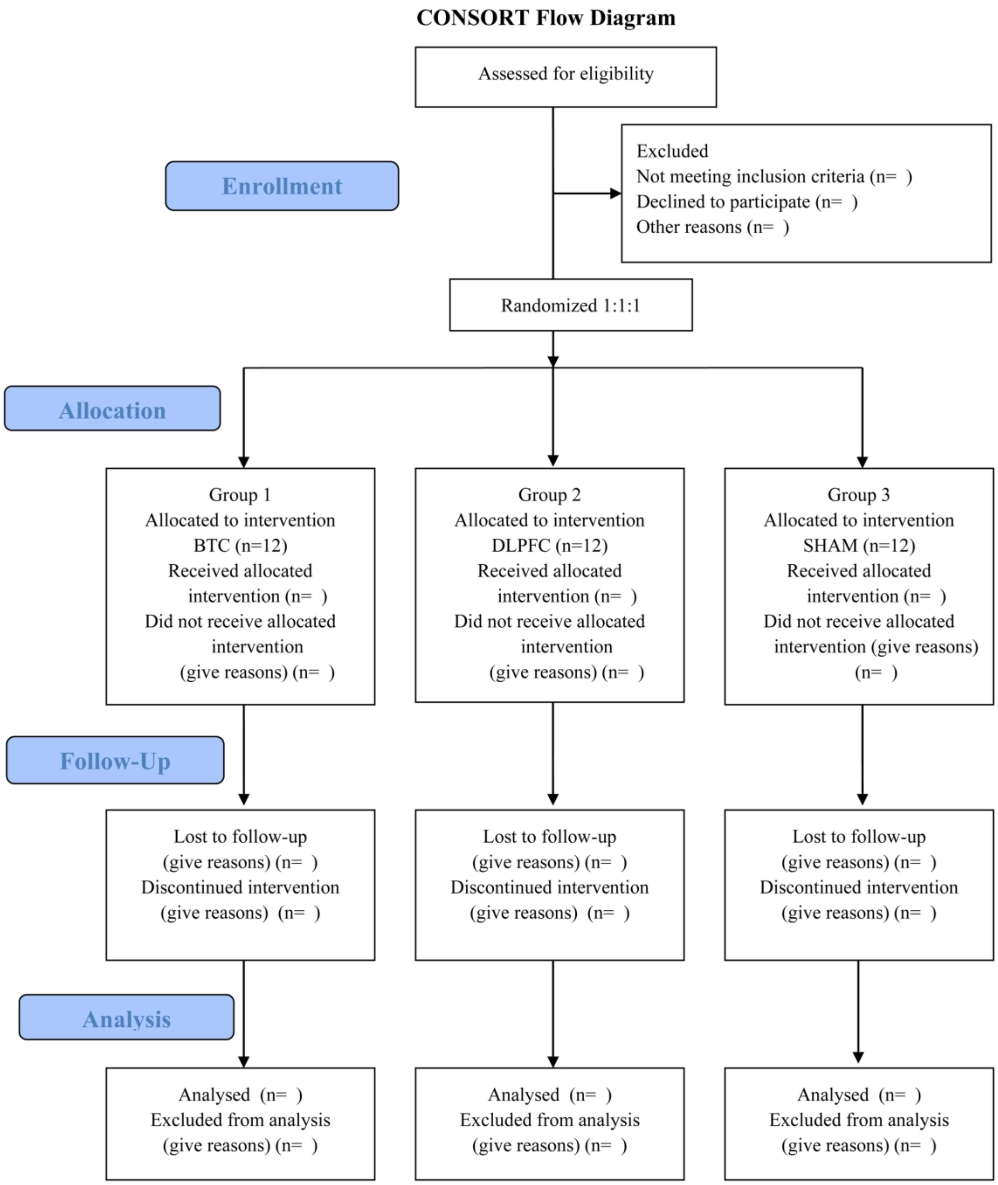


Figure 1 CONSORT Flow Diagram

184x219mm (300 x 300 DPI)

# CONSORT 2010 checklist of information to include when reporting a randomised trial*

| Section/Topic | Item No | Checklist item: The effects of transcranial direct current stimulation (tDCS) and cognitive training on episodic memory in patients with traumatic brain injury: a double-blind, randomized, placebo-controlled study | Reported on page No |
|---|---|---|---|
| **Title and abstract** | | | |
| | 1a | Identification as a randomised trial in the title | 1 |
| | 1b | Structured summary of trial design, methods, results, and conclusions (for specific guidance see CONSORT for abstracts) | 2 |
| **Introduction** | | | |
| Background and objectives | 2a | Scientific background and explanation of rationale | 4-6 |
| | 2b | Specific objectives or hypotheses | 6 |
| **Methods** | | | |
| Trial design | 3a | Description of trial design (such as parallel, factorial) including allocation ratio | 6 |
| | 3b | Important changes to methods after trial commencement (such as eligibility criteria), with reasons | n/a |
| Participants | 4a | Eligibility criteria for participants | 8 |
| | 4b | Settings and locations where the data were collected | 6 |
| Interventions | 5 | The interventions for each group with sufficient details to allow replication, including how and when they were actually administered | 10-11 |
| Outcomes | 6a | Completely defined pre-specified primary and secondary outcome measures, including how and when they were assessed | 9-10 |
| | 6b | Any changes to trial outcomes after the trial commenced, with reasons | n/a |
| Sample size | 7a | How sample size was determined | 11-12 |
| | 7b | When applicable, explanation of any interim analyses and stopping guidelines | n/a |
| Randomisation: | | | |
| Sequence generation | 8a | Method used to generate the random allocation sequence | 7 |
| | 8b | Type of randomisation; details of any restriction (such as blocking and block size) | 7 |
| Allocation concealment mechanism | 9 | Mechanism used to implement the random allocation sequence (such as sequentially numbered containers), describing any steps taken to conceal the sequence until interventions were assigned | 7 |
| Implementation | 10 | Who generated the random allocation sequence, who enrolled participants, and who assigned participants to interventions | 7 |



For peer review only - http://bmjopen.bmj.com/site/about/guidelines.xhtml

| | | | |
|---|---|---|---|
| Blinding | 11a | If done, who was blinded after assignment to interventions (for example, participants, care providers, those assessing outcomes) and how | 7 |
| | 11b | If relevant, description of the similarity of interventions | 7 |
| Statistical methods | 12a | Statistical methods used to compare groups for primary and secondary outcomes | 12 |
| | 12b | Methods for additional analyses, such as subgroup analyses and adjusted analyses | n/a |
| **Results** | | | |
| Participant flow (a diagram is strongly recommended) | 13a | For each group, the numbers of participants who were randomly assigned, received intended treatment, and were analysed for the primary outcome | n/a |
| | 13b | For each group, losses and exclusions after randomisation, together with reasons | n/a |
| Recruitment | 14a | Dates defining the periods of recruitment and follow-up | n/a |
| | 14b | Why the trial ended or was stopped | n/a |
| Baseline data | 15 | A table showing baseline demographic and clinical characteristics for each group | n/a |
| Numbers analysed | 16 | For each group, number of participants (denominator) included in each analysis and whether the analysis was by original assigned groups | n/a |
| Outcomes and estimation | 17a | For each primary and secondary outcome, results for each group, and the estimated effect size and its precision (such as 95% confidence interval) | n/a |
| | 17b | For binary outcomes, presentation of both absolute and relative effect sizes is recommended | n/a |
| Ancillary analyses | 18 | Results of any other analyses performed, including subgroup analyses and adjusted analyses, distinguishing pre-specified from exploratory | n/a |
| Harms | 19 | All important harms or unintended effects in each group (for specific guidance see CONSORT for harms) | n/a |
| **Discussion** | | | |
| Limitations | 20 | Trial limitations, addressing sources of potential bias, imprecision, and, if relevant, multiplicity of analyses | 14 |
| Generalisability | 21 | Generalisability (external validity, applicability) of the trial findings | 14 |
| Interpretation | 22 | Interpretation consistent with results, balancing benefits and harms, and considering other relevant evidence | n/a |
| **Other information** | | | |
| Registration | 23 | Registration number and name of trial registry | 15 |
| Protocol | 24 | Where the full trial protocol can be accessed, if available | 15 |
| Funding | 25 | Sources of funding and other support (such as supply of drugs), role of funders | 16 |

*We strongly recommend reading this statement in conjunction with the CONSORT 2010 Explanation and Elaboration for important clarifications on all the items. If relevant, we also recommend reading CONSORT extensions for cluster randomised trials, non-inferiority and equivalence trials, non-pharmacological treatments, herbal interventions, and pragmatic trials. Additional extensions are forthcoming: for those and for up to date references relevant to this checklist, see www.consort-statement.org.


For peer review only - http://bmjopen.bmj.com/site/about/guidelines.xhtml

# Reporting checklist for protocol of a clinical trial.

Based on the SPIRIT guidelines.

## Instructions to authors

Complete this checklist by entering the page numbers from your manuscript where readers will find each of the items listed below.

Your article may not currently address all the items on the checklist. Please modify your text to include the missing information. If you are certain that an item does not apply, please write "n/a" and provide a short explanation.

Upload your completed checklist as an extra file when you submit to a journal.

In your methods section, say that you used the SPIRITreporting guidelines, and cite them as:

Chan A-W, Tetzlaff JM, Altman DG, Laupacis A, Gøtzsche PC, Krleža-Jerić K, Hróbjartsson A, Mann H, Dickersin K, Berlin J, Doré C, Parulekar W, Summerskill W, Groves T, Schulz K, Sox H, Rockhold FW, Rennie D, Moher D. SPIRIT 2013 Statement: Defining standard protocol items for clinical trials. Ann Intern Med. 2013;158(3):200-207

| | | Reporting Item | Page Number |
|---|---|---|---|
| **Administrative information** | | | |
| Title | #1 | Descriptive title identifying the study design, population, interventions, and, if applicable, trial acronym | 1 |

For peer review only

| | | | |
|---|---|---|---|
| Trial registration | #2a | Trial identifier and registry name. If not yet registered, name of intended registry | 2 |
| Trial registration: data set | #2b | All items from the World Health Organization Trial Registration Data Set | 14 |
| Protocol version | #3 | Date and version identifier | n/a |
| Funding | #4 | Sources and types of financial, material, and other support | 15 |
| Roles and responsibilities: contributorship | #5a | Names, affiliations, and roles of protocol contributors | 15 |
| Roles and responsibilities: sponsor contact information | #5b | Name and contact information for the trial sponsor | n/a |
| Roles and responsibilities: sponsor and funder | #5c | Role of study sponsor and funders, if any, in study design; collection, management, analysis, and interpretation of data; writing of the report; and the decision to submit the report for publication, including whether they will have ultimate authority over any of these activities | 15 |
| Roles and responsibilities: committees | #5d | Composition, roles, and responsibilities of the coordinating centre, steering committee, endpoint adjudication committee, data management team, and other individuals or groups overseeing the trial, if applicable (see Item 21a for data monitoring committee) | 7 |

For peer review only

**Introduction**

| | | | |
|---|---|---|---|
| Background and rationale | #6a | Description of research question and justification for undertaking the trial, including summary of relevant studies (published and unpublished) examining benefits and harms for each intervention | 4-6 |
| Background and rationale: choice of comparators | #6b | Explanation for choice of comparators | 4-6 |
| Objectives | #7 | Specific objectives or hypotheses | 6 |
| Trial design | #8 | Description of trial design including type of trial (eg, parallel group, crossover, factorial, single group), allocation ratio, and framework (eg, superiority, equivalence, non-inferiority, exploratory) | 6 |

**Methods: Participants, interventions, and outcomes**

| | | | |
|---|---|---|---|
| Study setting | #9 | Description of study settings (eg, community clinic, academic hospital) and list of countries where data will be collected. Reference to where list of study sites can be obtained | 7 |
| Eligibility criteria | #10 | Inclusion and exclusion criteria for participants. If applicable, eligibility criteria for study centres and | 7-8 |

For peer review only

| | | | |
|---|---|---|---|
| | | individuals who will perform the interventions (eg, surgeons, psychotherapists) | |
| Interventions: description | #11a | Interventions for each group with sufficient detail to allow replication, including how and when they will be administered | 10-11 |
| Interventions: modifications | #11b | Criteria for discontinuing or modifying allocated interventions for a given trial participant (eg, drug dose change in response to harms, participant request, or improving / worsening disease) | n/a |
| Interventions: adherance | #11c | Strategies to improve adherence to intervention protocols, and any procedures for monitoring adherence (eg, drug tablet return; laboratory tests) | n/a |
| Interventions: concomitant care | #11d | Relevant concomitant care and interventions that are permitted or prohibited during the trial | n/a |
| Outcomes | #12 | Primary, secondary, and other outcomes, including the specific measurement variable (eg, systolic blood pressure), analysis metric (eg, change from baseline, final value, time to event), method of aggregation (eg, median, proportion), and time point for each outcome. Explanation of the clinical relevance of chosen efficacy and harm outcomes is strongly recommended | 9 |
| Participant timeline | #13 | Time schedule of enrolment, interventions (including any run-ins and washouts), assessments, and visits for participants. A schematic diagram is highly recommended | 8 |

For peer review only

| | | | |
|---|---|---|---|
| | | (see Figure) | |
| Sample size | #14 | Estimated number of participants needed to achieve study objectives and how it was determined, including clinical and statistical assumptions supporting any sample size calculations | 11 |
| Recruitment | #15 | Strategies for achieving adequate participant enrolment to reach target sample size | 7 |
| **Methods: Assignment of interventions (for controlled trials)** | | | |
| Allocation: sequence generation | #16a | Method of generating the allocation sequence (eg, computer-generated random numbers), and list of any factors for stratification. To reduce predictability of a random sequence, details of any planned restriction (eg, blocking) should be provided in a separate document that is unavailable to those who enrol participants or assign interventions | 7 |
| Allocation concealment mechanism | #16b | Mechanism of implementing the allocation sequence (eg, central telephone; sequentially numbered, opaque, sealed envelopes), describing any steps to conceal the sequence until interventions are assigned | 7 |
| Allocation: implementation | #16c | Who will generate the allocation sequence, who will enrol participants, and who will assign participants to interventions | 7 |

For peer review only

| | | | |
|---|---|---|---|
| Blinding (masking) | #17a | Who will be blinded after assignment to interventions (eg, trial participants, care providers, outcome assessors, data analysts), and how | 7 |
| Blinding (masking): emergency unblinding | #17b | If blinded, circumstances under which unblinding is permissible, and procedure for revealing a participant's allocated intervention during the trial | 7 |
| **Methods: Data collection, management, and analysis** | | | |
| Data collection plan | #18a | Plans for assessment and collection of outcome, baseline, and other trial data, including any related processes to promote data quality (eg, duplicate measurements, training of assessors) and a description of study instruments (eg, questionnaires, laboratory tests) along with their reliability and validity, if known. Reference to where data collection forms can be found, if not in the protocol | 9 |
| Data collection plan: retention | #18b | Plans to promote participant retention and complete follow-up, including list of any outcome data to be collected for participants who discontinue or deviate from intervention protocols | n/a |
| Data management | #19 | Plans for data entry, coding, security, and storage, including any related processes to promote data quality (eg, double data entry; range checks for data values). | 12 |

For peer review only

| | | | |
|---|---|---|---|
| | | Reference to where details of data management procedures can be found, if not in the protocol | |
| Statistics: outcomes | #20a | Statistical methods for analysing primary and secondary outcomes. Reference to where other details of the statistical analysis plan can be found, if not in the protocol | 11 |
| Statistics: additional analyses | #20b | Methods for any additional analyses (eg, subgroup and adjusted analyses) | 11 |
| Statistics: analysis population and missing data | #20c | Definition of analysis population relating to protocol non-adherence (eg, as randomised analysis), and any statistical methods to handle missing data (eg, multiple imputation) | 11 |
| **Methods: Monitoring** | | | |
| Data monitoring: formal committee | #21a | Composition of data monitoring committee (DMC); summary of its role and reporting structure; statement of whether it is independent from the sponsor and competing interests; and reference to where further details about its charter can be found, if not in the protocol. Alternatively, an explanation of why a DMC is not needed | 7 |
| Data monitoring: interim analysis | #21b | Description of any interim analyses and stopping guidelines, including who will have access to these interim results and make the final decision to terminate the trial | n/a |
| Harms | #22 | Plans for collecting, assessing, reporting, and managing solicited and spontaneously reported adverse events and other unintended effects of trial interventions or trial | 12 |

For peer review only

| | | | |
|---|---|---|---|
| | | conduct | |
| Auditing | #23 | Frequency and procedures for auditing trial conduct, if any, and whether the process will be independent from investigators and the sponsor | 7 |
| **Ethics and dissemination** | | | |
| Research ethics approval | #24 | Plans for seeking research ethics committee / institutional review board (REC / IRB) approval | 7 |
| Protocol amendments | #25 | Plans for communicating important protocol modifications (eg, changes to eligibility criteria, outcomes, analyses) to relevant parties (eg, investigators, REC / IRBs, trial participants, trial registries, journals, regulators) | n/a |
| Consent or assent | #26a | Who will obtain informed consent or assent from potential trial participants or authorised surrogates, and how (see Item 32) | 15 |
| Consent or assent: ancillary studies | #26b | Additional consent provisions for collection and use of participant data and biological specimens in ancillary studies, if applicable | n/a |
| Confidentiality | #27 | How personal information about potential and enrolled participants will be collected, shared, and maintained in order to protect confidentiality before, during, and after the trial | 12 |
| Declaration of | #28 | Financial and other competing interests for principal | 15 |

For peer review only

| | | | |
|---|---|---|---|
| interests | | investigators for the overall trial and each study site | |
| Data access | #29 | Statement of who will have access to the final trial dataset, and disclosure of contractual agreements that limit such access for investigators | 15 |
| Ancillary and post trial care | #30 | Provisions, if any, for ancillary and post-trial care, and for compensation to those who suffer harm from trial participation | n/a |
| Dissemination policy: trial results | #31a | Plans for investigators and sponsor to communicate trial results to participants, healthcare professionals, the public, and other relevant groups (eg, via publication, reporting in results databases, or other data sharing arrangements), including any publication restrictions | 14 |
| Dissemination policy: authorship | #31b | Authorship eligibility guidelines and any intended use of professional writers | n/a |
| Dissemination policy: reproducible research | #31c | Plans, if any, for granting public access to the full protocol, participant-level dataset, and statistical code | 15 |
| **Appendices** | | | |
| Informed consent materials | #32 | Model consent form and other related documentation given to participants and authorised surrogates | n/a |
| Biological specimens | #33 | Plans for collection, laboratory evaluation, and storage of biological specimens for genetic or molecular analysis in the current trial and for future use in ancillary studies, if applicable | n/a |

For peer review only

None The SPIRIT checklist is distributed under the terms of the Creative Commons Attribution License CC-BY-ND 3.0. This checklist can be completed online using https://www.goodreports.org/, a tool made by the EQUATOR Network in collaboration with Penelope.ai

For peer review only
